# Neutrosophic New Odd Weibull-Weibull Distribution with Heart Pulse Count Data Application

**DOI:** 10.12688/f1000research.172591.1

**Published:** 2026-02-10

**Authors:** Nooruldeen A. Noori, Mundher A. Khaleel, Raghad W. Faris

**Affiliations:** 1 Mathematics, University of Fallujah, Al-Fallujah, Al Anbar Governorate, 31002, Iraq; 2 Mathematics, Tikrit University, Tikrit, Saladin Governorate, 34011, Iraq

**Keywords:** Neutrosophic logic, New odd weibull, NNOWW, Neutrosophic incomplete moments, Heart pulse count.

## Abstract

This paper presents a new probability distribution known as the “Neutrosophic New Odd Weibull-Weibull Distribution (NNOWW)” by combining the proposed New Odd Weibull-Weibull (NOWW) distribution with the neutrosophic logic (the direct method of this logic). The distribution is presented in two theoretical frameworks. The derivation of several main distribution functions, along with their properties, is presented, accompanied by both theoretical and graphical representations of these properties. Parameters are estimated using three different methods, with Monte Carlo simulations conducted for various sample sizes to determine the estimation efficiency and the most suitable sample size for applying the distribution to real data. An applied framework tests its efficiency on real data representing the heart rate of 50 patients. The model was developed within this logic framework to define truth, falsity, and uncertainty in the data. The model was compared with five other neutrosophic distributions using criteria such as AIC, BIC, CAIC, and HQIC. The results showed a clear superiority of the NNOWW model across all indicators, confirming its accuracy and ability to represent uncertain data. Analytical graphs support this superiority. This model is recommended for use in medical fields and for analyzing Neutrosophic data.

## Introduction

The statistical theory of continuous probability distributions has undergone radical development over the past century, moving from simple single-parameter models to families of flexible multi-parameter distributions. Classical continuous distributions data back to the 18th century with distributions such as uniform (Laplace, 1774) and normal (Gauss, 1809), but the 20th century witnessed a paradigm shift with the emergence of families of generalized distributions from these families, such as the flexible Burr-XG distribution, ^1^ exponentiated truncated inverse Weibull distribution, ^2^ hybrid odd exponential-Φ, ^3^ Alpha power transformation and exponentiated TX, ^4^ Family of Truncated Positive, ^5^ truncated Burr-G, ^6^ Sec-G class, ^7^ modified TX, ^8^ Odd Lomax-G, ^9^ Marshall-Olkin Family, ^10^ Sine π-power odd-G, ^11^ and generalized logarithmic–X ^12^.

This development has become evident, particularly with the emergence of new mathematical concepts that address uncertainty and ambiguity in data. In this context, neutrosophic logic, introduced by Florentine, ^13, 14^ stands out as an advanced mathematical framework that extends beyond classical and fuzzy logic, offering a three-dimensional model consisting of truth (T), falsity (F), and uncertainty (I). ^15^ This approach has become an indispensable tool in analyzing data of an imprecise nature, such as those derived from medical measurements or observational studies, such as those derived from medical experiments or environmental measurements, where uncertainties are likely due to standard or methodological factors.

In the field of neutrosophic statistics, several probability distributions have been developed to handle interval-valued data. Examples of such distributions are (inverse Rayleigh distribution utilizing neutrosophic logic, ^16^ neutrosophic Generalized pareto, ^17^ Neutrosophic Burr XII, ^18^ Neutrosophic inverse power Lindley, ^19^ Neutrosophic exponentiated inverse Rayleigh, ^20^ Neutrosophic Topp-Leone, ^21^ Neutrosophic Topp-Leone Extended Exponential, ^22^ Neutrosophic Odd Lomax Generalized Exponential, ^23, 24^ Neutrosophic Gompertz Chen, ^25^ and Neutrosophic Beta-Lindley ^26^). However, there remains an urgent need for more flexible distributions that can model complex phenomena, especially in the medical and engineering fields. Therefore, this study presents a new distribution called the Neutrosophic New Odd Weibull-Weibull distribution (NNOWW), which combines the properties of the Weibull and the new odd Weibull family within this logic.

The proposed distribution offers a comprehensive probability model that incorporates neutrosophic parameters, enabling a more accurate representation of data with lower and upper bounds. Essential statistical properties such as the cumulative function, density function, hazard function, and moments are extracted and generalized to the neutrosophic environment.

The model is applied to real medical data (Heart rates data), which are often characterized by significant variability due to physiological factors or measurement errors. Its performance is compared with other neutrosophic distributions using criteria AIC, BIC, Kolmogorov-Smirnov tests, and other tests, demonstrating its superiority in capturing the variance in the data.

In Section 2 of the study, the neutrosophic cumulative and density distribution function (NCDF, NPDF) of NNOWW are derived in a neutrosophic environment. In Section 3, the statistical properties and moments are derived. In Section 4, the performance is estimated using three estimation methods, following a numerical simulation in Section 5. In Section 6, a practical application is performed on neutrosophic heart rate data, with the results compared to competing distributions. The study concludes by highlighting the efficiency of NNOWW in modeling fuzzy data, while suggesting future research directions, such as the application of machine learning in survival data analysis.

## Neutrosophic new odd Weibull-Weibull distribution

The New Odd Weibull-Weibull Distribution (NOWW) can be found by: The CDF and PDF of the New Odd Weibull-G family are defined for a positive random variable, X∈(0,∞) . The functions are defined respectively by the formulas ^27^:

$$F_{\text{NOWW}}\left(x,\lambda ,\delta \right)=1-e^{-\lambda {\left[-M\left(x\right).\log\left(1-M\left(x\right)\right)\right]}^{\delta }},x\;\geq \;0,\lambda ,\delta >0$$ (1)

$$f\left(x\right)=\frac{\delta \;m\left(x\right)\left[\frac{M\left(x\right)}{1-M\left(x\right)}-\log\left(1-M\left(x\right)\right)\right]}{{\left[-M\left(x\right).\log\left(1-M\left(x\right)\right)\right]}^{1-\delta }}e^{-\lambda {\left[-M\left(x\right).\log\left(1-M\left(x\right)\right)\right]}^{\delta }}$$ (2)

where M(x),m(x) are the CDF and PDF of any baseline distribution of the random variable X and λ,δ are shape parameters.

The CDF and PDF of the New Weibull distribution are defined for a random variable, X∈(0,∞) . The functions are defined respectively by the formulas ^28^:

$$M\left(x\right)=1-e^{-\alpha x^{\beta }},x\;\geq \;0,\alpha ,\beta >0$$ (3)

$$m\left(x\right)=\alpha \beta x^{\beta -1}e^{-\alpha x^{\beta }}$$ (4)

To find the NOWW, combine Equations 3 and 4 with Equations 1 and 2 to get the CDF and PDF for NOWW, respectively, in the form:

$$F_{\text{NOWW}}\left(x\right)=1-e^{-\lambda {\left[-\left(1-e^{-\alpha x^{\beta }}\right).\log\left(e^{-\alpha x^{\beta }}\right)\right]}^{\delta }}$$ (5)

$$f_{\text{NOWW}}\left(x\right)=\frac{\delta \alpha \beta x^{\beta -1}e^{-\alpha x^{\beta }}\left[\frac{1-e^{-\alpha x^{\beta }}}{e^{-\alpha x^{\beta }}}-\log\left(e^{-\alpha x^{\beta }}\right)\right]}{{\left[-\left(1-e^{-\alpha x^{\beta }}\right)\;\log\left(e^{-\alpha x^{\beta }}\right)\right]}^{1-\delta }}e^{-\lambda {\left[-\left(1-e^{-\alpha x^{\beta }}\right)\;\log\left(e^{-\alpha x^{\beta }}\right)\right]}^{\delta }}$$ (6)

The Neutrosophic shape parameters define the density (NPDF) of NNOWW $\lambda_{N},\delta_{N},\alpha_{N}$ and $\beta_{N}$ , which are within the ranges $\lambda_{N}\;\in \;\left[\lambda_{L},\lambda_{U}\right]$ , $\delta_{N}\;\in \;\left[\delta_{L},\delta_{U}\right]$ , $\alpha_{N}\;\in \;\left[\alpha_{L},\alpha_{U}\right]$ and $\beta_{N}\;\in \;\left[\beta_{L},\beta_{U}\right]$ respectively. The expression can be presented by updating the parameters and random variable in Equations 5 and 6 to neutrosophic parameters and neutrosophic random variable as follows:

$$F_{\text{NNOWW}}\left(x_{N}\right)=1-e^{-\lambda_{N}{\left[-\left(1-e^{-\alpha_{N}x_{N}^{\beta_{N}}}\right).\log\left(e^{-\alpha_{N}x_{N}^{\beta_{N}}}\right)\right]}^{\delta_{N}}}$$ (7)

$$\begin{array}{c} f_{\text{NNOWW}}\left(x_{N}\right)=\frac{\delta_{N}\alpha_{N}\beta_{N}x_{N}^{\beta_{N}-1}e^{-\alpha_{N}x_{N}^{\beta_{N}}}\left[\frac{1-e^{-\alpha_{N}x_{N}^{\beta_{N}}}}{e^{-\alpha_{N}x_{N}^{\beta_{N}}}}-\log\left(e^{-\alpha_{N}x_{N}^{\beta_{N}}}\right)\right]}{{\left[-\left(1-e^{-\alpha_{N}x_{N}^{\beta_{N}}}\right)\;\log\left(e^{-\alpha_{N}x_{N}^{\beta_{N}}}\right)\right]}^{1-\delta_{N}}}\\ \times e^{-\lambda_{N}{\left[-\left(1-e^{-\alpha_{N}x_{N}^{\beta_{N}}}\right).\log\left(e^{-\alpha_{N}x_{N}^{\beta_{N}}}\right)\right]}^{\delta_{N}}} \end{array}$$ (8)

**Figure 1.**
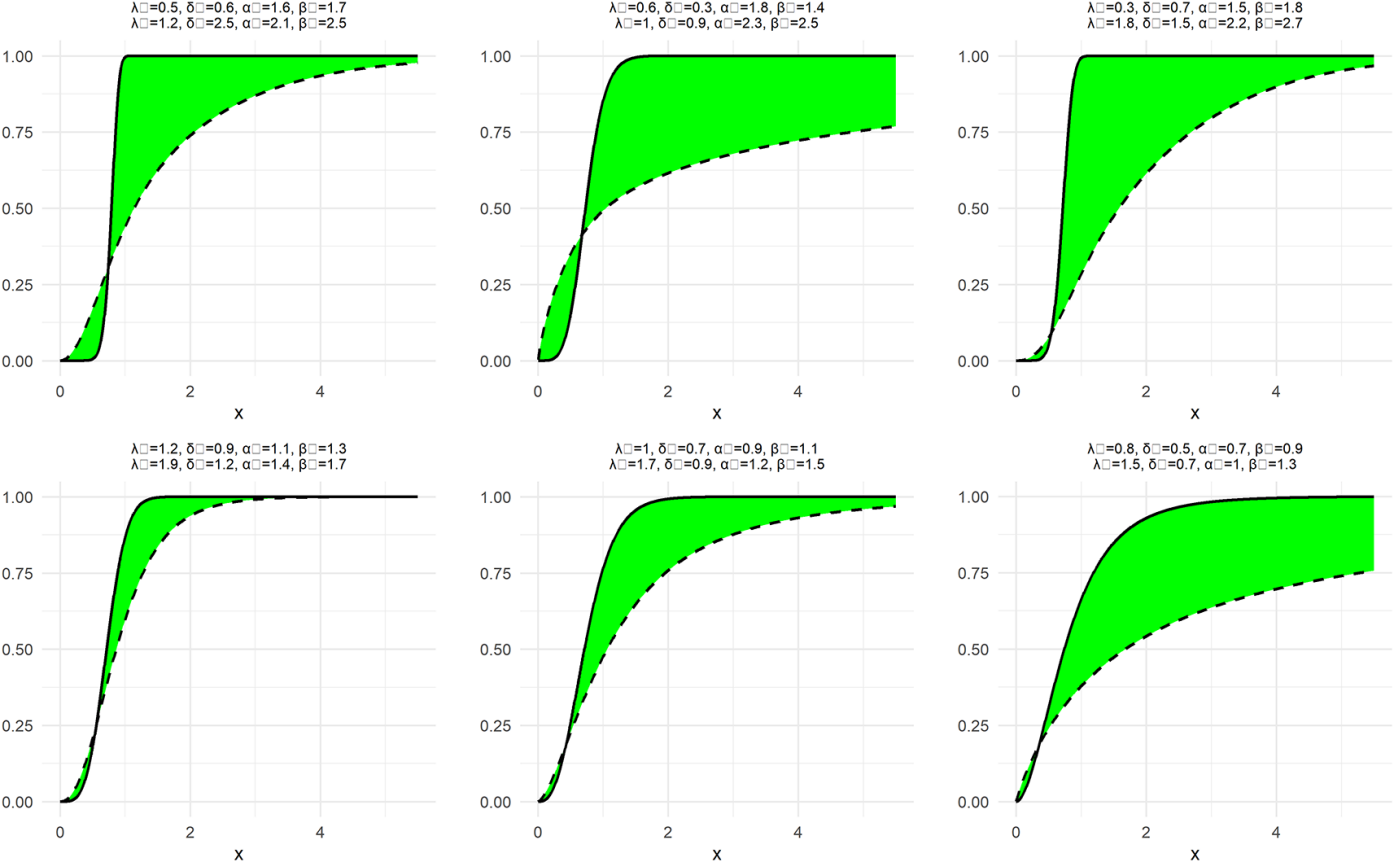
The NCDF function of NNOWW with different intervals.

**Figure 2.**
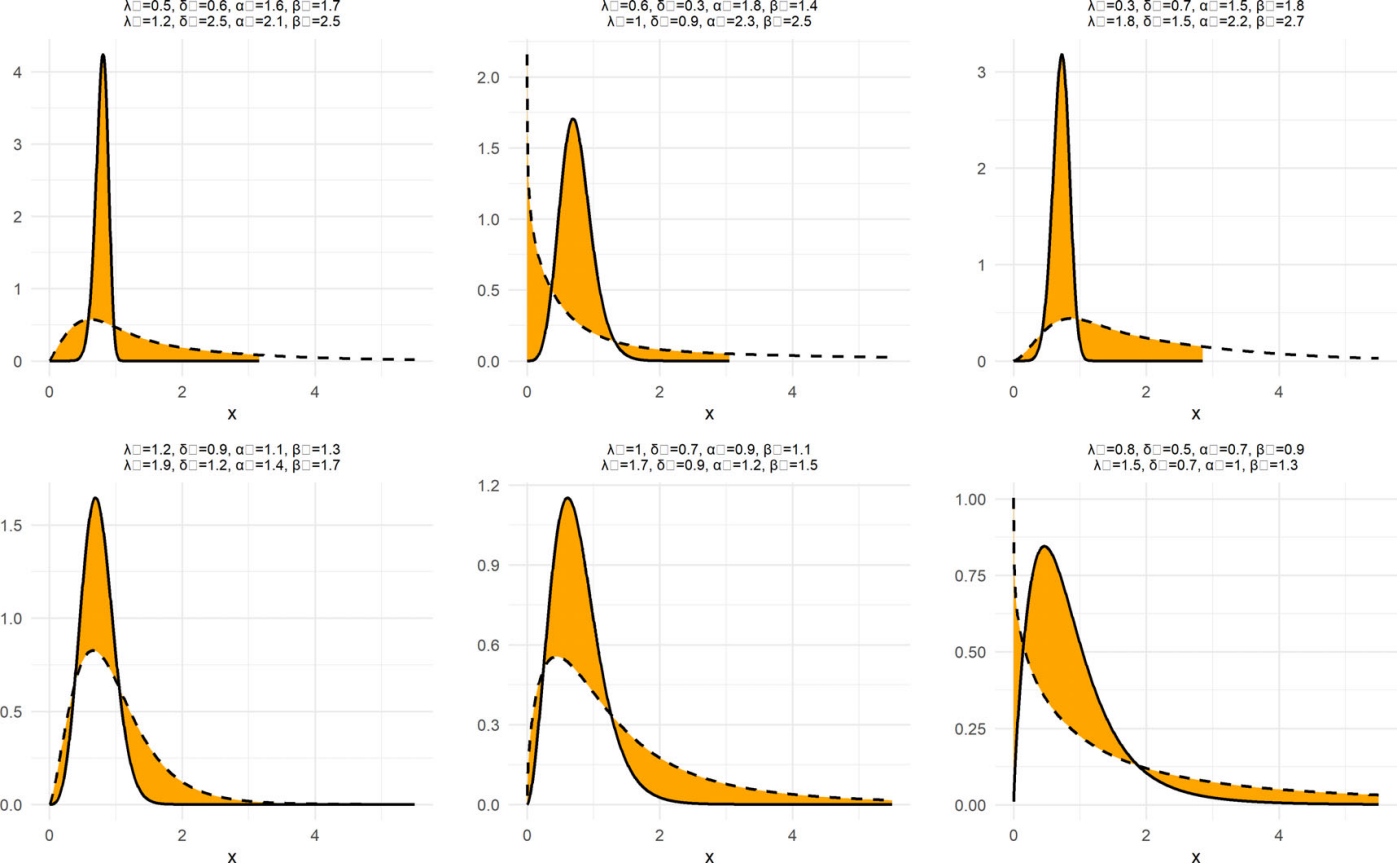
NPDF function of NNOWW with different intervals.

To demonstrate the nature and extent of the elasticity of NNOWW, the NCDF and NPDF functions shown in Equations 7 and 8 are plotted in Figures 1 and 2 with six different intervals for the neutrosophic parameters.

Figure 1 illustrates the behavior of the NCDF for the NNOWW distribution when the parameters are changed over different intervals. It can be observed that the function begins at zero and gradually increases until it reaches one, which is consistent with the fundamental properties of the cumulative distribution function. When the neutrosophic parameters are changed, the shape of the curve changes significantly. For example, as the parameter values increase, the curve becomes steeper, indicating that the data tends to cluster near specific values. In contrast, when the parameters are smaller, the curve is flatter, reflecting a more diffuse distribution of the data. This demonstrates the flexibility of the distribution in representing uncertain data, as the parameters can be adjusted to reflect varying degrees of uncertainty.

Figure 2 displays the NPDF for the NNOWW distribution under the influence of parameter variations. The curves exhibit a variety of shapes, ranging from long-tailed curves to curves more concentrated around the mean values. As the shape parameters ( $\delta_{N},\lambda_{N}$ ) as the increase continues, the curve becomes steeper and narrower, indicating a greater concentration of data around the mean values. Lower values of these parameters result in flatter, more spread-out curves, reflecting greater variability in the data. This flexibility makes the distribution suitable for modeling data with a high degree of uncertainty, such as medical or environmental data.

The neutrosophic survival function of NNOWW is given by the form ^23, 29^:

$$S_{\text{NNOWW}}\left(x_{N}\right)=e^{-\lambda_{N}{\left[-\left(1-e^{-\alpha_{N}x_{N}^{\beta_{N}}}\right).\log\left(e^{-\alpha_{N}x_{N}^{\beta_{N}}}\right)\right]}^{\delta_{N}}}$$ (9)

**Figure 3.**
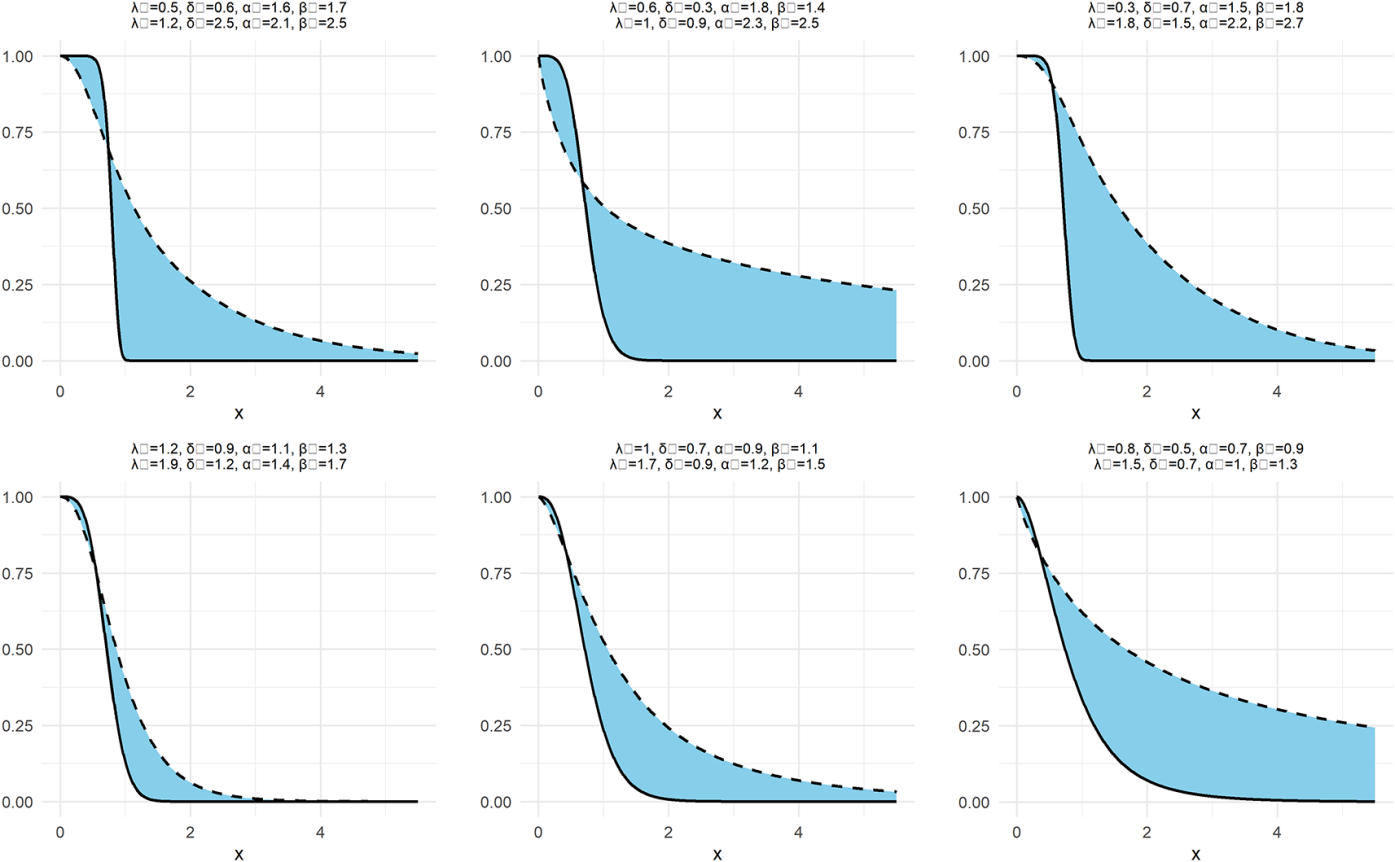
Neutrosophic survival function of NNOWW with different intervals.

In the same way as Figures 1 and 2 are drawn, above function in Figure 3 is also drawn for NNOWW as follows:

Figure 3 illustrates the neutrosophic survival function, which represents the probability of values remaining above a specific value. Analyzing the curves, we observe that the survival function gradually decreases as the values increase, a behavior expected in survival analysis. Changing the neutrosophic parameters affects the rate of decrease. For example, larger parameters cause the function to decrease more rapidly, indicating that the data tends to be concentrated within a smaller range of values. Conversely, smaller parameters lead to a slower decrease, reflecting a wider spread of the data. This highlights the distribution’s ability to represent data with different characteristics, making it a powerful tool for analyzing uncertain data.

The neutrosophic hazard function of NNOWW is given by the form as in ^16, 30^:

$$h_{\text{NNOWW}}\left(x_{N}\right)=\frac{\delta_{N}\alpha_{N}\beta_{N}x_{N}^{\beta_{N}-1}e^{-\alpha_{N}x_{N}^{\beta_{N}}}\left[\frac{1-e^{-\alpha_{N}x_{N}^{\beta_{N}}}}{e^{-\alpha_{N}x_{N}^{\beta_{N}}}}-\log\left(e^{-\alpha_{N}x_{N}^{\beta_{N}}}\right)\right]}{{\left[-\left(1-e^{-\alpha_{N}x_{N}^{\beta_{N}}}\right)\;\log\left(e^{-\alpha_{N}x_{N}^{\beta_{N}}}\right)\right]}^{1-\delta_{N}}}$$ (10)

## Mathematical properties of NNOWW

### Useful representations CDF, $\mathrm{CD}F^{\theta_{N}}$ , and pdf

The NCDF and NPDF functions, presented in Equations (7) and (8), respectively, are of great importance; however, this form makes integration, differentiation, and statistical analysis difficult. Therefore, these functions are expanded using power, exponential, and logarithmic series. This is because the original form of the functions contains very complex expressions that do not allow for a direct closed-form formula. Simplifying the mathematical operations and obtaining a flexible representation facilitates numerical analysis and application in estimation, calibration, and modeling. Therefore, the NCDF for NNOWW is expanded to the form:

$$F_{\text{NNOWW}}\left(x_{N}\right)=1-\varpi e^{-\alpha_{N}q_{N}x_{N}^{\beta_{N}}}$$ (11)

where $\varpi =\sum_{i,j,q=0}^{\infty }\frac{{\left(-1\right)}^{i_{N}+\delta_{N}+j_{N}}}{i_{N}!}d_{i_{N}\delta_{N},j_{N}}\lambda_{N}^{i_{N}}$ and $d_{i_{N}\delta_{N},j_{N}}=j_{N}^{-1}\sum_{w_{N}=1}^{j_{N}}\frac{\left[w_{N}\left(i_{N}\delta_{N}+1\right)-j_{N}\right]}{w_{N}+1}$ for $j_{N}\;\geq \;0$ and $d_{i_{N}k_{N},0}=1$

The $F_{\text{NNOWW}}^{\theta_{N}}\left(x_{N}\right)$ has a form:

$$F_{\text{NNOWW}}^{\theta_{N}}\left(x_{N}\right)={\left(1-e^{-\lambda_{N}{\left[-\left(1-e^{-\alpha_{N}x_{N}^{\beta_{N}}}\right).\log\left(e^{-\alpha_{N}x_{N}^{\beta_{N}}}\right)\right]}^{\delta_{N}}}\right)}^{\theta_{N}}$$ (12)

In the same way, it can be expanded to form:

$$F_{\text{NNOWW}}^{\theta_{N}}\left(x_{N}\right)=\Theta e^{-\alpha_{N}v_{N}x_{N}^{\beta_{N}}}$$ (13)

Where

Θ=∑qN,lN,vN=0∞(−1)qN+lN+δN+uN+vNlN!(θNqN)(uN+2lNδNvN)dlNδN,uNλNlNqNlN,dlNδN,uN=uN−1∑sN=1uN[sN(lNδN+1)−uN]sN+1foruN≥0

And

$$d_{l_{N}k_{N},0}=1$$

The NPDF for NNOWW can be expanded into the following series form:

$$f_{\text{NNOWW}}\left(x_{N}\right)=\psi x_{N}^{\beta_{N}-1}e^{-\alpha_{N}\left(1+p_{N}\right)x_{N}^{\beta_{N}}}-\varphi x_{N}^{\beta_{N}-1}e^{-\alpha_{N}\left(1+t_{N}\right)x_{N}^{\beta_{N}}}$$ (14)

Where

ψ=∑iN,kN,nN,pN=0∞(−1)iN+2δN+kN+nN+pN−1iN!(kN+2iNδN+2δN+nN−1pN)diNδN+δN−1,kNλNiN+1δNαNβN

φ=∑iN,jN,tN=0∞(−1)iN+2δN+jN+tN−1iN!(jN+2iNδN+2δN−1tN)diNδN+δN,jNλNiN+1δNαNβN

such as

$$d_{i_{N}\delta_{N}+\delta_{N}-1,k_{N}}=k_{N}^{-1}\sum_{s_{N}=1}^{k_{N}}\frac{\left[s_{N}\left(i_{N}\delta_{N}+\delta_{N}-1+1\right)-k_{N}\right]}{s_{N}+1}$$

for $k_{N}\;\geq \;0$ and $d_{i_{N}\delta_{N}+\delta_{N}-1,0}=1$ , and $d_{i_{N}\delta_{N}+\delta_{N},j_{N}}=j_{N}^{-1}\sum_{l_{N}=1}^{j_{N}}\frac{\left[l_{N}\left(i_{N}\delta_{N}+\delta_{N}+1\right)-j_{N}\right]}{l_{N}+1}$ for $j_{N}\;\geq \;0$ and $d_{i_{N}\delta_{N}+\delta_{N},0}=1$

### Neutrosophic quantile function

The Neutrosophic quantile function (NQF) utilizes quantiles to determine the normal limits of values and detect extreme values within a distribution. It represents the mathematical inverse of the NCDF function $F_{\text{NNOWW}}\left(x_{N}\right)$ , and is known as ^31^:

$$Q\left(c\right)=F_{\text{NNOWW}}^{-1}\left(x_{N}\right),c\;\in \;\left(0,1\right)$$

That is, it gives the value $x_{N}$ at which the probability that the random variable $X_{N}$ is less than or equal to $x_{N}$ is $c_{N}$ . In other words:

$$P\left(X_{N}\;\leq \;Q\left(c\right)\right)=c$$

Then the NQF for NNOWW has a form:

$$Q\left(c\right)={\left[\frac{\log\left(\frac{B}{B+W_{-1}Be^{B}}\right)}{\alpha_{N}}\right]}^{\frac{1}{\beta_{N}}},B={\left(-\frac{\log\left(1-p_{N}\right)}{\lambda_{N}}\right)}^{\frac{1}{\delta_{N}}}$$ (15)

where $W_{-1}$ is the lower Lambert W function.

The Table 1 shows the results of NQF for the NNOWW distribution across four sets of parameters (Set-1 to Set-4) shown below:

**Table 1.** The NQF for NNOWW.

c	Set-1	Set-2	Set-3	Set-4
0.1	[0.45228, 0.55995]	[0.50515, 0.59244]	[0.59131, 0.63519]	[0.46689, 0.57397]
0.2	[0.54255, 0.59969]	[0.59840, 0.63264]	[0.67374,0.68183]	[0.55260, 0.61288]
0.3	[0.61042, 0.62657]	[0.65979,0.66774]	[0.69962,0.74758]	[0.61614, 0.63917]
0.4	[0.64841,0.66993]	[0.68180,0.72809]	[0.72046,0.80379]	[0.66041,0.67124]
0.5	[0.66788,0.72664]	[0.70140,0.78523]	[0.73899,0.85627]	[0.67933,0.72323]
0.6	[0.68649,0.78427]	[0.72011,0.84309]	[0.75661,0.90860]	[0.69739,0.77565]
0.7	[0.70547,0.84695]	[0.73920,0.90565]	[0.77454,0.96440]	[0.71578,0.83210]
0.8	[0.72661,0.92157]	[0.76045,0.97986]	[0.79439,1.02956]	[0.73622,0.89870]
0.9	[0.75414,1.02736]	[0.78808,1.08459]	[0.82014,1.11969]	[0.76279,0.99193]

Set-1: $\lambda_{N}=\left[1.1,2.1\right],\delta_{N}=\left[1.6,2.6\right],\alpha_{N}=\left[1.8,2.8\right],\beta_{N}=\left[1.5,2.5\right]$

Set-2: $\lambda_{N}=\left[1.2,2.2\right],\delta_{N}=\left[1.5,2.5\right],\alpha_{N}=\left[1.6,2.6\right],\beta_{N}=\left[1.7,2.7\right]$

Set-3: $\lambda_{N}=\left[1.3,2.3\right],\delta_{N}=\left[1.6,2.6\right],\alpha_{N}=\left[1.4,2.4\right],\beta_{N}=\left[1.9,2.9\right]$

Set-4: $\lambda_{N}=\left[1.4,2.4\right],\delta_{N}=\left[1.6,2.6\right],\alpha_{N}=\left[1.7,2.7\right],\beta_{N}=\left[1.6,2.7\right]$

The results of Table 1 show how Q(c) values change at nine probability levels ranging from (0.1 to 0.9). Each result is given as an interval [lower, higher] representing the quantile’s neutrosophic values, where the lower limit reflects the degree of “truth” in the probability and the upper limit reflects the degree of “falseness” or vice versa, depending on the logical interpretation in a neutrosophic setting.

Comparing the values across the four sets, we note that set-3 produces the highest quantile values at each. c level, while set-1 produces the lowest. This suggests that the parameters in set-3 (remarkably the low $\alpha_{N}$ and the high $\beta_{N}$ ) Result in a distribution that is more concentrated toward higher values; that is, the neutrosophic random variable tends to take larger values. In contrast, set-1 reflects a distribution more centered around smaller values.

The differences between the lower and higher values in each interval also reflect the amount of uncertainty in the probability estimation, which is the essence of the neutrosophic approach. The larger the gap between the two limits, the greater the degree of uncertainty. From this, we note that the differences are greater at the higher quantile values (0.8, 0.9), indicating that the extreme values carry the most significant amount of ambiguity.

### Neutrosophic moments

Neutrosophic Moments function (NMF) are an extension of classical moments in statistics, taking into account degree of uncertainty. Their mathematical definition for a neutrosophic random variable $X_{N}$ , of order r , is ^25, 32, 33^:

$${\mu_{N}}_{r}=E\left(x_{N}^{r}\right)=\int_{-\infty }^{\infty }x_{N}^{r}f\left(x_{N}\right)dx_{N}$$ (16)

Then the NMF for NNOWW has a form:

$${\mu_{N}}_{r}=\psi \int_{0}^{\infty }x_{N}^{\beta_{N}+r-1}e^{-\alpha_{N}\left(1+p_{N}\right)x_{N}^{\beta_{N}}}dx_{N}-\varphi \int_{0}^{\infty }x_{N}^{\beta_{N}+r-1}e^{-\alpha_{N}\left(1+t_{N}\right)x_{N}^{\beta_{N}}}dx_{N}$$

By integrate above equation to get a final form:

$${\mu_{N}}_{r}=\frac{\Gamma \left(\frac{\beta_{N}+r}{\beta_{N}}\right)}{\beta_{N}}\left[\frac{\psi }{{\left\{\alpha_{N}\left(1+p_{N}\right)\right\}}^{\frac{\beta_{N}+r}{\beta_{N}}}}-\frac{\varphi }{{\left\{\alpha_{N}\left(1+t_{N}\right)\right\}}^{\frac{\beta_{N}+r}{\beta_{N}}}}\right]$$ (17)

Then the first four NMF for NNOWW are:

$${\mu_{N}}_{1}=\frac{\Gamma \left(\frac{\beta_{N}+1}{\beta_{N}}\right)}{\beta_{N}}\left[\frac{\psi }{{\left\{\alpha_{N}\left(1+p_{N}\right)\right\}}^{\frac{\beta_{N}+1}{\beta_{N}}}}-\frac{\varphi }{{\left\{\alpha_{N}\left(1+t_{N}\right)\right\}}^{\frac{\beta_{N}+1}{\beta_{N}}}}\right]$$ (18)

$${\mu_{N}}_{2}=\frac{\Gamma \left(\frac{\beta_{N}+2}{\beta_{N}}\right)}{\beta_{N}}\left[\frac{\psi }{{\left\{\alpha_{N}\left(1+p_{N}\right)\right\}}^{\frac{\beta_{N}+2}{\beta_{N}}}}-\frac{\varphi }{{\left\{\alpha_{N}\left(1+t_{N}\right)\right\}}^{\frac{\beta_{N}+2}{\beta_{N}}}}\right]$$ (19)

$${\mu_{N}}_{3}=\frac{\Gamma \left(\frac{\beta_{N}+3}{\beta_{N}}\right)}{\beta_{N}}\left[\frac{\psi }{{\left\{\alpha_{N}\left(1+p_{N}\right)\right\}}^{\frac{\beta_{N}+3}{\beta_{N}}}}-\frac{\varphi }{{\left\{\alpha_{N}\left(1+t_{N}\right)\right\}}^{\frac{\beta_{N}+3}{\beta_{N}}}}\right]$$ (20)

$${\mu_{N}}_{4}=\frac{\Gamma \left(\frac{\beta_{N}+4}{\beta_{N}}\right)}{\beta_{N}}\left[\frac{\psi }{{\left\{\alpha_{N}\left(1+p_{N}\right)\right\}}^{\frac{\beta_{N}+4}{\beta_{N}}}}-\frac{\varphi }{{\left\{\alpha_{N}\left(1+t_{N}\right)\right\}}^{\frac{\beta_{N}+4}{\beta_{N}}}}\right]$$ (21)

From it, we can get the neutrosophic variance ( $\sigma_{N}^{2}$ ),neutrosophic skewness ( $\gamma_{1_{N}}$ ) and neutrosophic kurtosis ( $\gamma_{2_{N}}$ ) of the NNOWW distribution as follows:

$$\begin{array}{c} \sigma_{N}^{2}=\frac{\Gamma \left(\frac{\beta_{N}+2}{\beta_{N}}\right)}{\beta_{N}}\left[\frac{\psi }{{\left\{\alpha_{N}\left(1+p_{N}\right)\right\}}^{\frac{\beta_{N}+2}{\beta_{N}}}}-\frac{\varphi }{{\left\{\alpha_{N}\left(1+t_{N}\right)\right\}}^{\frac{\beta_{N}+2}{\beta_{N}}}}\right]\\ -{\left(\frac{\Gamma \left(\frac{\beta_{N}+1}{\beta_{N}}\right)}{\beta_{N}}\left[\frac{\psi }{{\left\{\alpha_{N}\left(1+p_{N}\right)\right\}}^{\frac{\beta_{N}+1}{\beta_{N}}}}-\frac{\varphi }{{\left\{\alpha_{N}\left(1+t_{N}\right)\right\}}^{\frac{\beta_{N}+1}{\beta_{N}}}}\right]\right)}^{2} \end{array}$$ (22)

$$\gamma_{1_{N}}=\frac{\frac{\Gamma \left(\frac{\beta_{N}+3}{\beta_{N}}\right)}{\beta_{N}}\left[\frac{\psi }{{\left\{\alpha_{N}\left(1+p_{N}\right)\right\}}^{\frac{\beta_{N}+3}{\beta_{N}}}}-\frac{\varphi }{{\left\{\alpha_{N}\left(1+t_{N}\right)\right\}}^{\frac{\beta_{N}+3}{\beta_{N}}}}\right]}{{\left(\frac{\Gamma \left(\frac{\beta_{N}+2}{\beta_{N}}\right)}{\beta_{N}}\left[\frac{\psi }{{\left\{\alpha_{N}\left(1+p_{N}\right)\right\}}^{\frac{\beta_{N}+2}{\beta_{N}}}}-\frac{\varphi }{{\left\{\alpha_{N}\left(1+t_{N}\right)\right\}}^{\frac{\beta_{N}+2}{\beta_{N}}}}\right]\right)}^{\frac{3}{2}}}$$ (23)

$$\gamma_{2_{N}}=\frac{\frac{\Gamma \left(\frac{\beta_{N}+4}{\beta_{N}}\right)}{\beta_{N}}\left[\frac{\psi }{{\left\{\alpha_{N}\left(1+p_{N}\right)\right\}}^{\frac{\beta_{N}+4}{\beta_{N}}}}-\frac{\varphi }{{\left\{\alpha_{N}\left(1+t_{N}\right)\right\}}^{\frac{\beta_{N}+4}{\beta_{N}}}}\right]}{{\left(\frac{\Gamma \left(\frac{\beta_{N}+2}{\beta_{N}}\right)}{\beta_{N}}\left[\frac{\psi }{{\left\{\alpha_{N}\left(1+p_{N}\right)\right\}}^{\frac{\beta_{N}+2}{\beta_{N}}}}-\frac{\varphi }{{\left\{\alpha_{N}\left(1+t_{N}\right)\right\}}^{\frac{\beta_{N}+2}{\beta_{N}}}}\right]\right)}^{2}}-3$$ (24)

Table 2 displays the first four NMF of the NNOWW distribution across different combinations of neutrosophic parameter values, neutrosophic variance, neutrosophic skewness, and neutrosophic kurtosis.

**Table 2.** Some NMF for NNOWW with some different parameter intervals.

$\lambda_{N}$	$\delta_{N}$	$\alpha_{N}$	$\beta_{N}$	${\mu_{N}}_{1}$	${\mu_{N}}_{2}$	${\mu_{N}}_{3}$	${\mu_{N}}_{4}$	$\sigma_{N}^{2}$	$\gamma_{1_{N}}$	$\gamma_{2_{N}}$
[0.3,1.3]	[0.5,1.5]	[0.4, 1.4]	[0.1,1.1]	[0.16542, 0.361763]	[0.049509, 0.206052]	[0.018051, 0.137492]	[0.007425,0.100571]	[0.022145, 0.075179]	[1.638594, 1.469984]	[3.029336, 2.368754]
			[0.2,1.2]	[0.302855, 0.366473]	[0.166756, 0.215811]	[0.109175, 0.147791]	[0.078795,0.110347]	[0.075035, 0.081509]	[1.603256, 1.474136]	[2.833598, 2.369253]
		[0.6, 1.6]	[0.3,1.3]	[0.352472, 0.407897]	[0.194587, 0.244091]	[0.126835, 0.168749]	[0.091043,0.126761]	[0.07035, 0.077711]	[1.477648, 1.399306]	[2.404482, 2.127568]
			[0.4,1.4]	[0.35999, 0.405622]	[0.219826, 0.248172]	[0.155074, 0.17452]	[0.118573,0.132862]	[0.090233, 0.083643]	[1.504603, 1.411613]	[2.45374, 2.157226]
	[0.7,1.7]	[0.7, 1.7]	[0.5,1.5]	[0.34727, 0.399294]	[0.204115, 0.242399]	[0.140075, 0.169511]	[0.104921,0.128511]	[0.083519, 0.082963]	[1.518964, 1.420375]	[2.51832, 2.187154]
			[0.6,1.6]	[0.337936, 0.394968]	[0.207571, 0.243836]	[0.14714, 0.172704]	[0.112966,0.132236]	[0.09337, 0.087836]	[1.555895, 1.434357]	[2.621883, 2.224111]
		[0.9,1.9]	[0.7,1.7]	[0.389739, 0.43127]	[0.241025, 0.270162]	[0.171081, 0.193058]	[0.13126, 0.148696]	[0.089128, 0.084167]	[1.445799, 1.374837]	[2.259477, 2.037279]
			[0.8,1.8]	[0.370871, 0.422638]	[0.235029, 0.267888]	[0.169866, 0.193149]	[0.132141,0.149802]	[0.097483, 0.089265]	[1.490822, 1.393035]	[2.392188, 2.087428]

The Table 2 includes values for the intervals [lower, upper] that represent the moments of the distribution in an inexact setting, taking uncertainty into account. From the table, it is noted that increasing the parameters (especially $\beta_{N}$ and $\delta_{N}$ ) leads to a change in the shape of the distribution, compressing it around the mean or spreading it out, depending on whether the values are small or large. Higher values of the moments reflect greater skewness in the data, while lower values indicate concentration around the median value.

Also, it is noted that the differences between the lower and upper bounds of each moment are wider when the probability values are high or when the parameters are distributed over larger intervals, indicating greater uncertainty in the estimate. This is consistent with the nature of the neutrosophic approach, where each probability value is expressed as a range rather than a single value, providing greater flexibility for modeling uncertain medical or environmental data.

**Figure 4.**
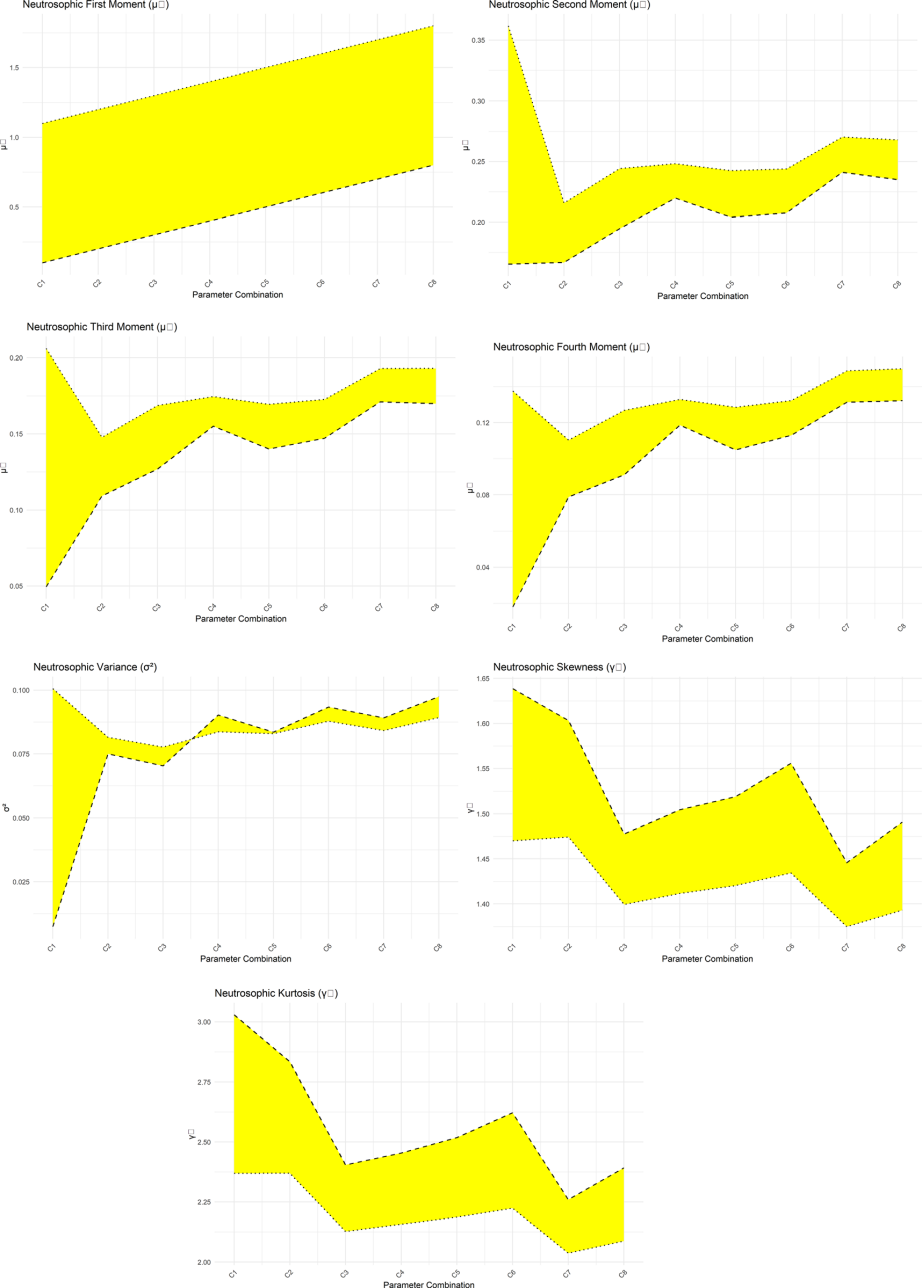
NMF of NNOWW with different intervals.

Figure 4 presents a graphical representation of the NNOWW distribution’s NMF s across multiple parameter intervals. The figure illustrates how the neutrosophic values change at specific moments as the parameters are adjusted. The curves representing these moments define the significant impact of the parameters on the nature of the distribution.

The figure clearly shows that some intervals result in a more compressed distribution, meaning that the values are concentrated around a single point. In contrast, other intervals result in a more diffuse distribution, reflecting a greater variability in the values. This confirms that the NNOWW model has the flexibility to adapt to different data characteristics, especially data that is inherently noisy or fuzzy, such as the heartbeat data used in the application.

Table 3 presents the minimum and maximum NMF extracted from all analyzed intervals.

**Table 3.** Extracting the minimum and maximum values of neutrosophic.

Parameter	Min	Max	Average interval
${\mu_{N}}_{1}$	0.100000	1.800000	1.00000000
${\mu_{N}}_{2}$	0.165420	0.361763	0.05747413
${\mu_{N}}_{3}$	0.049509	0.206052	0.04459738
${\mu_{N}}_{4}$	0.018051	0.149802	0.03486963
$\sigma_{N}^{2}$	0.007425	0.100571	0.01014025
$\gamma_{1_{N}}$	1.445799	1.474136	-0.10724225
$\gamma_{2_{N}}$	2.259477	2.369253	-0.36928137

The Table 3 a quick numerical summary of the levels of uncertainty and skewness across NNOWW. It is clear that the differences between the extremes (lower and upper) gradually decrease at higher points, indicating that uncertainty is greater at the early points (the mean) and gradually decreases at higher points. The decrease in the value of the differences between the extremes indicates that the distribution is more stable in terms of its shape, skewness, and kurtosis, while retaining some flexibility in the location of the mean and variance.

The neutrosophic moment generating function (N.M.G.F) of any neutrosophic random variable $X_{N}$ is defined mathematically by the formula ^34, 35^:

$$M_{x_{N}}\left(t_{N}\right)=E\left(e^{t_{N}x_{N}}\right)=\int_{-\infty }^{\infty }e^{t_{N}x_{N}}f\left(x_{N}\right)dx_{N}$$

Using exponential expansion for above equation, then we get a form:

$$M_{x_{N}}\left(t_{N}\right)=\sum_{s_{N}=0}^{\infty }\frac{t_{N}^{s_{N}}}{s_{N}!}\left[{\mu_{N}}_{r}\right],r=1,2,3,4$$

Using Equation (17) to get a form:

$$M_{x_{N}}\left(t_{N}\right)=\sum_{s_{N}=0}^{\infty }\frac{t_{N}^{s_{N}}}{s_{N}!}\left[\frac{\Gamma \left(\frac{\beta_{N}+r}{\beta_{N}}\right)}{\beta_{N}}\left[\frac{\psi }{{\left\{\alpha_{N}\left(1+p_{N}\right)\right\}}^{\frac{\beta_{N}+r}{\beta_{N}}}}-\frac{\varphi }{{\left\{\alpha_{N}\left(1+t_{N}\right)\right\}}^{\frac{\beta_{N}+r}{\beta_{N}}}}\right]\right]$$ (25)

The neutrosophic Characteristic function (N.C.F) of any neutrosophic random variable $X_{N}$ is defined mathematically by the formula ^36^:

$$Q_{x_{N}}\left(t_{N}\right)=E\left(e^{it_{N}x_{N}}\right)=\int_{0}^{\infty }e^{it_{N}x_{N}}f\left(x_{N}\right)dx_{N}$$

Using exponential expansion for above equation, then we get a form:

$$Q_{x_{N}}\left(t_{N}\right)=\sum_{v_{N}=0}^{\infty }\frac{{\left(it_{N}\right)}^{v_{N}}}{v_{N}!}\left[{\mu_{N}}_{r}\right]$$

Using Equation (17) to get a form:

$$Q_{x_{N}}\left(t_{N}\right)=\sum_{v_{N}=0}^{\infty }\frac{{\left(it_{N}\right)}^{v_{N}}}{v_{N}!}\left[\frac{\Gamma \left(\frac{\beta_{N}+r}{\beta_{N}}\right)}{\beta_{N}}\left[\frac{\psi }{{\left\{\alpha_{N}\left(1+p_{N}\right)\right\}}^{\frac{\beta_{N}+r}{\beta_{N}}}}-\frac{\varphi }{{\left\{\alpha_{N}\left(1+t_{N}\right)\right\}}^{\frac{\beta_{N}+r}{\beta_{N}}}}\right]\right]$$ (26)

Neutrosophic Incomplete Moments are an extension of classical Incomplete moments in statistics, taking into account degree of uncertainty. Their mathematical definition for a neutrosophic random variable $X_{N}$ , of order r , is ^25, 32, 33^:

$$M_{r}\left(y\right)=\int_{-\infty }^{y}x_{N}^{r}f\left(x_{N}\right)dx_{N}$$ (27)

Then the Neutrosophic Incomplete Moments for NNOWW from above equation and Equation (14) has a form:

$$M_{r}\left(y\right)=\psi \int_{0}^{y}x_{N}^{\beta_{N}+r-1}e^{-\alpha_{N}\left(1+p_{N}\right)x_{N}^{\beta_{N}}}dx_{N}-\varphi \int_{0}^{y}x_{N}^{\beta_{N}+r-1}e^{-\alpha_{N}\left(1+t_{N}\right)x_{N}^{\beta_{N}}}dx_{N}$$

By integrate above equation to get a final form:

$$M_{r}\left(y\right)=\frac{1}{\beta_{N}}\left[\frac{\psi \Gamma \left(\frac{\beta_{N}+r}{\beta_{N}},\alpha_{N}\left(1+p_{N}\right)y^{\beta_{N}}\right)}{{\left\{\alpha_{N}\left(1+p_{N}\right)\right\}}^{\frac{\beta_{N}+r}{\beta_{N}}}}-\frac{\varphi \Gamma \left(\frac{\beta_{N}+r}{\beta_{N}},\alpha_{N}\left(1+t_{N}\right)y^{\beta_{N}}\right)}{{\left\{\alpha_{N}\left(1+t_{N}\right)\right\}}^{\frac{\beta_{N}+r}{\beta_{N}}}}\right]$$ (28)

### Neutrosophic Rényi entropy

The Neutrosophic Rényi entropy is a generalization of the Shannon entropy. It provides a parametric family of entropy measures that can be adjusted using a parameter $\theta_{N}$ , which controls the sensitivity to events with small probabilities. Let $f\left(x_{N}\right)$ be a NPDF for any distribution then the Neutrosophic Rényi entropy is defined as ^37^:

$$R_{\theta_{N}}\left(f\right)=\frac{1}{1-\theta_{N}}\log\left(\int_{0}^{\infty }f^{\theta_{N}}\left(x_{N}\right)dx_{N}\right),\theta_{N}>0\;\&\;\theta_{N}\neq 1$$ (29)

From Equations (29) and (14) we get:

$$R_{\theta_{N}}\left(f\right)=\frac{1}{1-\theta_{N}}\log\left(\int_{0}^{\infty }{\left(\psi x_{N}^{\beta_{N}-1}e^{-\alpha_{N}\left(1+p_{N}\right)x_{N}^{\beta_{N}}}-\varphi x_{N}^{\beta_{N}-1}e^{-\alpha_{N}\left(1+t_{N}\right)x_{N}^{\beta_{N}}}\right)}^{\theta_{N}}dx_{N}\right)$$

Using the Binomial Series

(ψxNβN−1e−αN(1+pN)xNβN−φxNβN−1e−αN(1+tN)xNβN)θN=∑k=0∞(−1)k(θNk)ψθN−kxNβN+θN−k−1e−αN(θN−k)(1+pN)xNβNφkxNβN+k−1e−αNk(1+tN)xNβN

$${\left(\psi x_{N}^{\beta_{N}-1}e^{-\alpha_{N}\left(1+p_{N}\right)x_{N}^{\beta_{N}}}-\varphi x_{N}^{\beta_{N}-1}e^{-\alpha_{N}\left(1+t_{N}\right)x_{N}^{\beta_{N}}}\right)}^{\theta_{N}}=\sum_{k=0}^{\infty }{\left(-1\right)}^{k}\left(\begin{array}{c} \theta_{N}\\ k \end{array}\right)\psi^{\theta_{N}-k}x_{N}^{2\beta_{N}+\theta_{N}-2}e^{-\alpha_{N}\left[\theta_{N}\left(1+p_{N}\right)+k\left(1+t_{N}\right)\right]x_{N}^{\beta_{N}}}\varphi^{k}$$

Then we get:

$$R_{\theta_{N}}\left(f\right)=\frac{1}{1-\theta_{N}}\log\left(\sum_{k=0}^{\infty }{\left(-1\right)}^{k}\left(\begin{array}{c} \theta_{N}\\ k \end{array}\right)\psi^{\theta_{N}-k}\varphi^{k}\int_{0}^{\infty }x_{N}^{2\beta_{N}+\theta_{N}-2}e^{-Wx_{N}^{\beta_{N}}}dx_{N}\right)$$

Where

$$W=\alpha_{N}\left[\theta_{N}\left(1+p_{N}\right)+k\left(1+t_{N}\right)\right]$$

the final form is:

$$R_{\theta_{N}}\left(f\right)=\frac{1}{1-\theta_{N}}\log\left(\sum_{k=0}^{\infty }{\left(-1\right)}^{k}\left(\begin{array}{c} \theta_{N}\\ k \end{array}\right)\psi^{\theta_{N}-k}\varphi^{k}\frac{1}{\beta_{N}}W^{-\frac{2\beta_{N}+\theta_{N}-1}{\beta_{N}}}\Gamma \left(\frac{\beta_{N}+\theta_{N}-1}{\beta_{N}}\right)\right)$$ (30)

## Estimation parameters for NNOWW

### Maximum likelihood estimation - MLE

Maximum Likelihood Estimation (MLE) is a fundamental inferential technique employed to estimate the unknown parameters of a statistical model. It operates by identifying the parameter values that maximize the likelihood function, which quantifies the probability of observing the given dataset under the assumed model structure.

Formally, consider a random sample $X_{N}=\left(x_{N1},x_{N2},\ldots ,x_{\mathrm{Nn}}\right)$ drawn from a population characterized by a probability density function $f\left(x_{N}|\gamma_{N}\right)$ , where $\gamma_{N}$ Denotes the vector of unknown parameters ( $\gamma_{N}=\left(\lambda_{N},\delta_{N},\alpha_{N},\beta_{N}\right)$ ). The likelihood function $L\left(\gamma_{N}|x_{N}\right)$ is then defined as ^38^:

$$L\left(\gamma_{N}|x_{N}\right)=\prod_{i=1}^{n}f\left(x_{\mathrm{Ni}}|\gamma_{N}\right)$$

From Equation 8 the MLE for NNOWW has a form:

$$\begin{array}{c} L\left(\gamma_{N}|x_{N}\right)=\prod_{i=1}^{n}\frac{\delta_{N}\alpha_{N}\beta_{N}x_{\mathrm{Ni}}^{\beta_{N}-1}e^{-\alpha_{N}x_{\mathrm{Ni}}^{\beta_{N}}}\left[\frac{1-e^{-\alpha_{N}x_{\mathrm{Ni}}^{\beta_{N}}}}{e^{-\alpha_{N}x_{\mathrm{Ni}}^{\beta_{N}}}}-\log\left(e^{-\alpha_{N}x_{\mathrm{Ni}}^{\beta_{N}}}\right)\right]}{{\left[-\left(1-e^{-\alpha_{N}x_{\mathrm{Ni}}^{\beta_{N}}}\right)\;\log\left(e^{-\alpha_{N}x_{\mathrm{Ni}}^{\beta_{N}}}\right)\right]}^{1-\delta_{N}}}\\ \times e^{-\lambda_{N}{\left[-\left(1-e^{-\alpha_{N}x_{\mathrm{Ni}}^{\beta_{N}}}\right).\log\left(e^{-\alpha_{N}x_{\mathrm{Ni}}^{\beta_{N}}}\right)\right]}^{\delta_{N}}} \end{array}$$

The objective of MLE is to find the parameter vector ${\hat{\gamma }}_{N}$ that maximizes this function, that is:

$${\hat{\gamma }}_{N}=\arg\max_{\gamma_{N}}L\left(\gamma_{N}|x_{N}\right)$$

In practice, for computational convenience, the natural logarithm of the likelihood function, known as the log-likelihood, is often used:

$$\begin{array}{c} l\left(\gamma_{N}|x_{N}\right)=\text{nlog}\left(\lambda_{N}\right)+\text{nlog}\left(\delta_{N}\right)+\text{nlog}\left(\alpha_{N}\right)+\text{nlog}\left(\beta_{N}\right)-\sum_{i=1}^{n}\alpha_{N}x_{\text{iN}}^{\beta_{N}}\\ +\left(\beta_{N}-1\right)\sum_{i=1}^{n}\log\left(x_{\mathrm{Ni}}\right)+\sum_{i=1}^{n}\log\left[\frac{1-e^{-\alpha_{N}x_{\text{iN}}^{\beta_{N}}}}{e^{-\alpha_{N}x_{\text{iN}}^{\beta_{N}}}}-\log\left(e^{-\alpha_{N}x_{\text{iN}}^{\beta_{N}}}\right)\right]\\ \begin{array}{c} +\left(\delta_{N}-1\right)\sum_{i=1}^{n}\log\left[-\left(1-e^{-\alpha_{N}x_{\text{iN}}^{\beta_{N}}}\right).\log\left(e^{-\alpha_{N}x_{\text{iN}}^{\beta_{N}}}\right)\right]\\ -\lambda_{N}\sum_{i=1}^{n}\left[-\left(1-e^{-\alpha_{N}x_{\text{iN}}^{\beta_{N}}}\right).\log\left(e^{-\alpha_{N}x_{\mathrm{Ni}}^{\beta_{N}}}\right)\right] \end{array} \end{array}$$ (31)

The estimation process involves solving the system of equations obtained by setting the partial derivatives of the log-likelihood function in Equation 31 concerning each parameter to zero. This yields the maximum likelihood estimates of $\gamma_{N}$ .

### Least squares estimation - LSE

Least Squares Estimation (LSE) is used to estimate the parameters of a linear regression model by minimizing the sum of the squared differences between the observed values and the values predicted by the model, and defined as:

$$\varphi \left(\gamma_{N}\right)=\sum_{i=1}^{n}{\left[F\left(x_{\mathrm{Ni}}\right)-\frac{i}{n+1}\right]}^{2}$$

Then the LSE for NNOWW form above equation and equation has a form:

$$\varphi \left(\gamma_{N}\right)=\sum_{i=1}^{n}{\left[1-e^{-\lambda_{N}{\left[-\left(1-e^{-\alpha_{N}x_{\mathrm{Ni}}^{\beta_{N}}}\right).\log\left(e^{-\alpha_{N}x_{\mathrm{Ni}}^{\beta_{N}}}\right)\right]}^{\delta_{N}}}-\frac{i}{n+1}\right]}^{2}$$ (32)

### Weighted least squares estimation - WLS

Weighted Least Squares (WLS) is a type of extension of LSE used when the assumption of constant variance (homoscedasticity) in the error terms is violated. It assigns weights to each observation to address heteroscedasticity. and defined as:

$$W\left(\gamma_{N}\right)=\sum_{i=1}^{n}\;W_{i}{\left[F\left(x_{\mathrm{Ni}}\right)-\frac{i}{n+1}\right]}^{2}$$

where $W_{i}$ is the weight to each observation defined by form:

$$W_{i}=\frac{{\left(n+1\right)}^{2}\left(n+2\right)}{i\left(n-i+1\right)}$$

Then the LSE for NNOWW form above equation and equation has a form:

$$W\left(\gamma_{N}\right)=\sum_{i=1}^{n}\;W_{i}{\left[1-e^{-\lambda_{N}{\left[-\left(1-e^{-\alpha_{N}x_{\mathrm{Ni}}^{\beta_{N}}}\right).\log\left(e^{-\alpha_{N}x_{\mathrm{Ni}}^{\beta_{N}}}\right)\right]}^{\delta_{N}}}-\frac{i}{n+1}\right]}^{2}$$ (33)

Equations 32 and 33 are solved in the same manner as Equation 31: by differentiating the four NNOWW parameters and then setting the derivatives to zero.

### Monte carlo simulation for NNOWW

Simulation is a fundamental step in evaluating the performance of statistical estimation methods under controlled experimental conditions. It allows for testing the accuracy, reliability, and efficiency of the various parameter estimators presented in Section 4 when they are repeatedly drawn from a known distribution with predetermined parameters.

In this study, a Monte Carlo simulation was conducted to evaluate the behavior of the proposed NNOWW using various sample sizes. The goal was to measure the ability of each estimation method to retrieve the actual values of the parameters when applied to randomly generated data, while simultaneously reflecting the nature of neutrosophic uncertainty.

Several performance indicators were used, including the arithmetic mean of the estimates to measure the average estimated values across iterations; bias ^39^ to measure the difference between the estimated and actual values; mean square error (MSE) ^40^ to combine the variance and bias of the estimate; and root mean square error (RMSE) ^41, 42^ to directly measure the average error in the estimate. The simulation was conducted for four different sample sizes (30, 60, 120, and 240 to 1000 iteration) to analyze and improve the effectiveness of the estimation methods as the data volume increases. Neutrosophic intervals were used for the parameters instead of exact values to represent the uncertain nature of the data, in line with the neutrosophic logic adopted in the study.

The results shown in Table 4 reflect the efficiency default parameter values are given to illustrate the estimation efficiency of each method and highlight the strengths and shortcomings of each, enhancing the reliability of the proposed distribution and providing a reliable scientific basis for future applications of ambiguous data, as shown below.

**Table 4.** Monte Carlo simulations conducted for the NNOWW.

$\lambda_{N}=\left[0.6,1.3\right],\;\delta_{N}=\left[0.9,1.5\right],\;\alpha_{N}=\left[1.3,2\right],\;\beta_{N}=\left[1.1,1.8\right]$					
N	Est.	Ess. Par.	MLE	LSE	WLSE
30	Mean	$\hat{\lambda_{N}}$	[0.4731169, 0.9417599]	[0.7711335, 1.4008241]	[0.7266131, 1.4021852]
		$\hat{\delta_{N}}$	[0.261129, 0.415340]	[0.91608361, 2.1374088]	[0.95818773, 1.8859218]
		$\hat{\alpha_{N}}$	[1.0567087, 1.5312916]	[1.32390424, 1.94378311]	[1.28140916, 1.90790076]
		$\hat{\beta_{N}}$	[1.5833194, 2.7764508]	[1.13869668, 1.76044053]	[1.098543767, 1.83897995]
	MSE	$\hat{\lambda_{N}}$	[0.1094562, 1.0299969]	[0.5930069, 1.8729167]	[0.6027220, 2.1474641]
		$\hat{\delta_{N}}$	[0.5138209, 2.175253]	[1.259348, 7.5610757]	[1.0961917, 5.4150269]
		$\hat{\alpha_{N}}$	[0.2215006, 0.5922265]	[0.24327905, 0.50033826]	[0.20523242, 0.39752342]
		$\hat{\beta_{N}}$	[0.3280687, 1.3600469]	[0.25775167, 0.79524709]	[0.149488418,0.7763517]
	RMSE	$\hat{\lambda_{N}}$	[0.3308416, 1.0148876]	[0.7700694, 1.3685455]	[0.59513606, 1.4654228]
		$\hat{\delta_{N}}$	[0.716813, 1.474874]	[1.12220675, 2.7497410]	[1.04699174, 2.3270210]
		$\hat{\alpha_{N}}$	[0.4706385, 0.7695625]	[0.49323326, 0.70734593]	[0.45302585, 0.63049459]
		$\hat{\beta_{N}}$	[0.5727728, 1.1662105]	[0.5076925, 0.89176628]	[0.386637321, 0.77145062]
	Bias	$\hat{\lambda_{N}}$	[0.1268831, 0.3582401]	[0.1008241,0.1711335]	[0.1021852,0.1266131]
		$\hat{\delta_{N}}$	[0.638871, 1.084660]	[0.01608361, 0.6374088]	[0.05818773, 0.3859218]
		$\hat{\alpha_{N}}$	[0.2432913, 0.4687084]	[0.02390424, 0.05621689]	[0.01859084, 0.09209924]
		$\hat{\beta_{N}}$	[0.4833194, 0.9764508]	[0.03869668, 0.03955947]	[0.001456233, 0.03897995]
60	Mean	$\hat{\lambda_{N}}$	[0.4579281, 1.0079291]	[0.67632673, 1.0663367]	[0.66410287, 1.2051158]
		$\hat{\delta_{N}}$	[0.4448405, 0.7878873]	[0.92906412, 2.2690161]	[0.7413881, 2.1133648]
		$\hat{\alpha_{N}}$	[1.0182885, 1.6597458]	[1.301502163, 1.8277665]	[1.309694413, 1.8977473]
		$\hat{\beta_{N}}$	[1.4637667, 2.4619177]	[1.102333232, 1.6643533]	[1.2024717, 1.75863942]
	MSE	$\hat{\lambda_{N}}$	[0.150517, 0.5735284]	[0.26513797, 0.5197256]	[0.08807544, 0.6670142]
		$\hat{\delta_{N}}$	[0.7419072, 1.5855009]	[0.58664515, 5.2526605]	[0.3627973, 5.0659667]
		$\hat{\alpha_{N}}$	[0.2387491, 0.4035017]	[0.143078123, 0.2078617]	[0.074959938, 0.1798887]
		$\hat{\beta_{N}}$	[0.2051029, 0.8387267]	[0.096332162, 0.2744889]	[0.1038933, 0.33205186]
	RMSE	$\hat{\lambda_{N}}$	[0.3879652, 0.7573166]	[0.5149155, 0.72092]	[0.29677506, 0.8167094]
		$\hat{\delta_{N}}$	[0.8613403, 1.2591667]	[0.76592764, 2.2918683]	[0.6023266, 2.2507703]
		$\hat{\alpha_{N}}$	[0.4886195, 0.6352178]	[0.378256689, 0.4559185]	[0.273788126, 0.4241328]
		$\hat{\beta_{N}}$	[0.4528828, 0.9158203]	[0.31037423, 0.5239169]	[0.3223248, 0.57623941]
	Bias	$\hat{\lambda_{N}}$	[0.1420719, 0.2920709]	[0.07632673, 0.2336633]	[0.06410287, 0.0948842]
		$\hat{\delta_{N}}$	[0.4551595, 0.7121127]	[0.02906412, 0.7690161]	[0.1586119, 0.6133648]
		$\hat{\alpha_{N}}$	[0.2817115, 0.3402542]	[0.001502163, 0.1722335]	[0.009694413, 0.1022527]
		$\hat{\beta_{N}}$	[0.3637667, 0.6619177]	[0.002333232, 0.1356467]	[0.1024717, 0.04136058]
120	Mean	$\hat{\lambda_{N}}$	[0.4956312, 1.28096219]	[0.61370177, 1.1982902]	[0.607106756, 1.21684849]
		$\hat{\delta_{N}}$	[0.5967458, 1.2605198]	[0.94748995, 1.9717478]	[0.92980872, 1.6999761]
		$\hat{\alpha_{N}}$	[1.0629113, 1.8942291]	[1.28347878, 1.93024777]	[1.2719124, 1.91345542]
		$\hat{\beta_{N}}$	[1.3381724, 2.0695964]	[1.11236134, 1.70721706]	[1.13739539, 1.75941061]
	MSE	$\hat{\lambda_{N}}$	[0.1493804, 0.54959818]	[0.09070187, 0.2600601]	[0.056249846, 0.1932341]
		$\hat{\delta_{N}}$	[0.4906619, 0.9233345]	[0.47036258, 1.9803674]	[0.66663631, 0.6789318]
		$\hat{\alpha_{N}}$	[0.2000869, 0.2682699]	[0.06503452, 0.1021018]	[0.04047426, 0.05625788]
		$\hat{\beta_{N}}$	[0.1256648, 0.3111961]	[0.05262082, 0.13067612]	[0.05174358, 0.10445817]
	RMSE	$\hat{\lambda_{N}}$	[0.3864975, 0.7413489]	[0.30116752, 0.5099608]	[0.237170499, 0.43958401]
		$\hat{\delta_{N}}$	[0.7004727, 0.960903]	[0.68582985, 1.4072553]	[0.81647799, 0.8239732]
		$\hat{\alpha_{N}}$	[0.4473107, 0.5179478]	[0.25501867, 0.31953372]	[0.20118215, 0.23718743]
		$\hat{\beta_{N}}$	[0.3544923, 0.5578495]	[0.22939228, 0.36149152]	[0.22747215, 0.32319989]
	Bias	$\hat{\lambda_{N}}$	[0.01903781,0.1043688]	[0.01370177, 0.1017098]	[0.007106756, 0.08315151]
		$\hat{\delta_{N}}$	[0.2394802,0.3032542]	[0.04748995, 0.4717478]	[0.02980872, 0.1999761]
		$\hat{\alpha_{N}}$	[0.1057709,0.2370887]	[0.01652122, 0.06975223]	[0.0280876, 0.08654458]
		$\hat{\beta_{N}}$	[0.2381724, 0.2695964]	[0.01236134, 0.09278294]	[0.03739539, 0.04058939]
240	Mean	$\hat{\lambda_{N}}$	[0.54320063, 1.4009697]	[0.598018087, 1.33376588]	[0.61509583, 1.307017214]
		$\hat{\delta_{N}}$	[0.7044551, 1.48460863]	[0.908360368, 1.8495684]	[0.86719178, 1.7511264]
		$\hat{\alpha_{N}}$	[1.1345002, 1.95689457]	[1.28037007, 2.01286632]	[1.301495296, 2.01924748]
		$\hat{\beta_{N}}$	[1.25253026, 1.916726]	[1.105064356, 1.74370952]	[1.14134209, 1.77808693]
	MSE	$\hat{\lambda_{N}}$	[0.12666889, 0.5416475]	[0.049313203, 0.32798305]	[0.03725054, 0.122370597]
		$\hat{\delta_{N}}$	[0.3921219, 0.83107656]	[0.220488483, 1.3906743]	[0.22530915, 1.369072]
		$\hat{\alpha_{N}}$	[0.1670802, 0.2163778]	[0.04166935, 0.07196096]	[0.036487149, 0.03760822]
		$\hat{\beta_{N}}$	[0.07279197, 0.150986]	[0.02684792, 0.07757471]	[0.02311166, 0.05099818]
	RMSE	$\hat{\lambda_{N}}$	[0.35590573, 0.7359671]	[0.222065762, 0.57269804]	[0.19300399,0.47466741]
		$\hat{\delta_{N}}$	[0.6261964, 0.91163401]	[0.469562012, 1.1792686]	[0.34981509, 1.1700735]
		$\hat{\alpha_{N}}$	[0.4087545, 0.46516428]	[0.20413072, 0.26825539]	[0.191016097, 0.1939284]
		$\hat{\beta_{N}}$	[0.26979987, 0.3885691]	[0.163853349, 0.27852236]	[0.1520252, 0.22582776]
	Bias	$\hat{\lambda_{N}}$	[0.05679937, 0.1009697]	[0.001981913, 0.03376588]	[0.007017214,0.01509583]
		$\hat{\delta_{N}}$	[0.01539137,0.1955449]	[0.008360368, 0.3495684]	[0.2511264,0.03280822]
		$\hat{\alpha_{N}}$	[0.04310543,0.1654998]	[0.01286632,0.01962993]	[0.001495296, 0.01924748]
		$\hat{\beta_{N}}$	[0.116726,0.15253026]	[0.005064356, 0.05629048]	[0.02191307,0.04134209]

From Table 4, it is clear that.

• Size 30: At this small sample size, the estimates show wide variation among the three methods, especially in the MSE and RMSE metrics. The maximum likelihood method performs averagely, but the LSE and WLSE show greater variation in estimates, reflected in the high MSE values. LSE produces the highest bias, indicating that the method is unstable in small samples, and WLSE suffers from dispersion in estimates, especially in the second and third estimates. The significant difference between the lower and upper bounds for all methods reflects the high degree of uncertainty in the estimate at a small sample size.
• Size 60: As the sample size increases, the performance of the three methods improves, and the MSE and RMSE values decrease significantly compared to size 30, indicating greater stability in the estimates, as the MLE method still provides results that are more balanced between error and bias. LSE and WLSE still exhibit variation, but to a lesser extent than before. We observe that the bias gradually decreases, approaching zero in some estimates, especially in the third MLE estimation, indicating that the results are close to the actual values.
• Size 120: This size represents an intermediate stage and shows significantly improved performance across all indicators. There is a sharp decrease in MSE and RMSE, especially in the second and third parameter estimations. MLE continues to provide stable and low-error performance, with bias close to zero in most estimations. LSE and WLSE show significant improvements compared to the previous two sizes, especially in estimating the mean and deviation, but WLSE is still slightly affected by dispersion in some ranges.
• Size 240: At this large size, all methods approach optimal efficiency. MSE and RMSE values are at their lowest across all estimations, demonstrating high stability and accuracy. MLE outperforms in terms of bias reduction, especially in the first and second estimations. WLSE exhibits strong performance in estimating the second and third parameters but shows a slight weakness in the first parameter due to the wide gap between the lower and upper bounds, indicating some residual uncertainty in the estimation.

Thus, as the sample size increases, all methods improve; however, the MLE method is the most balanced in terms of reducing bias and variance across all stages.

**Figure 5.**
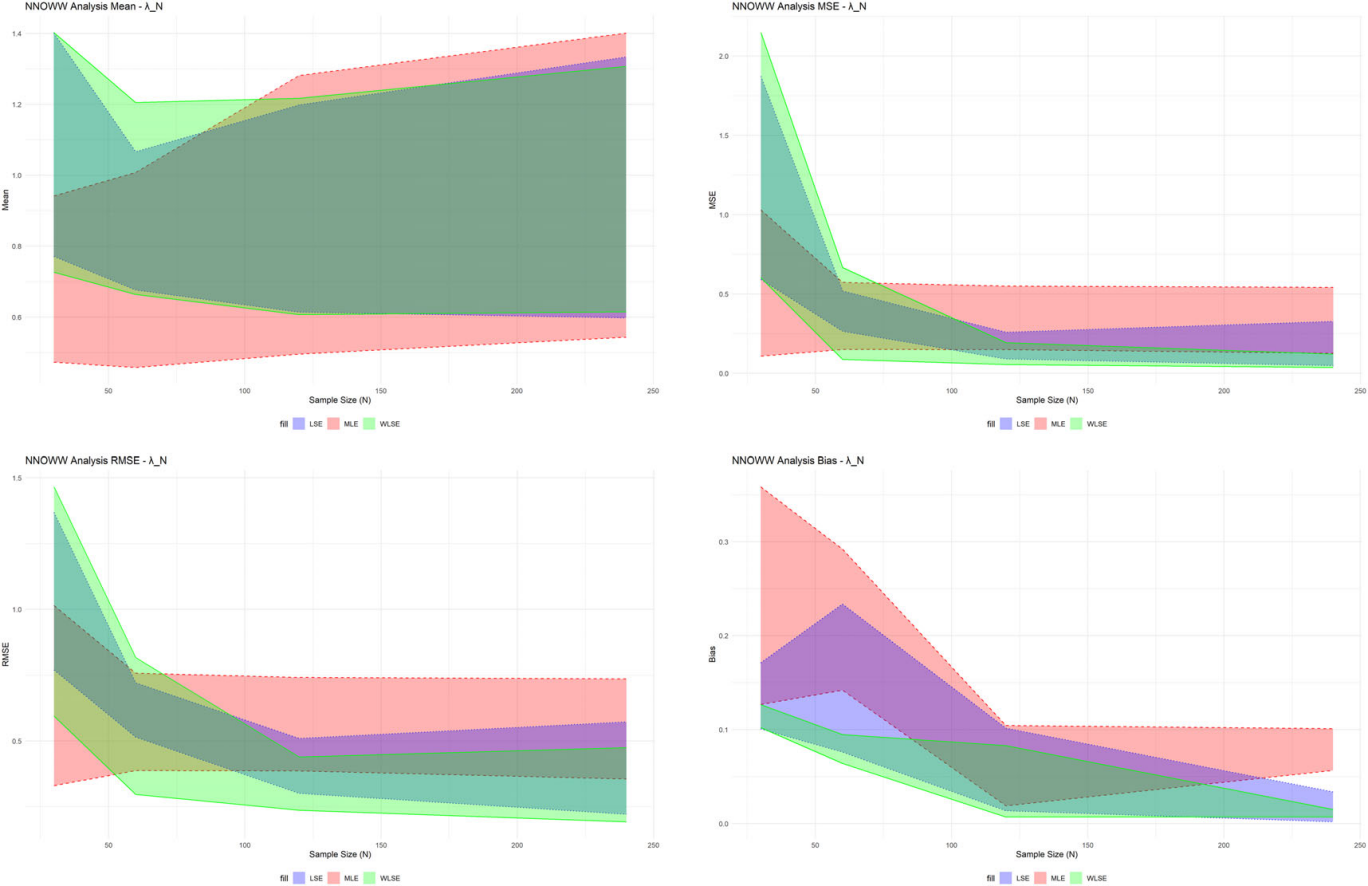

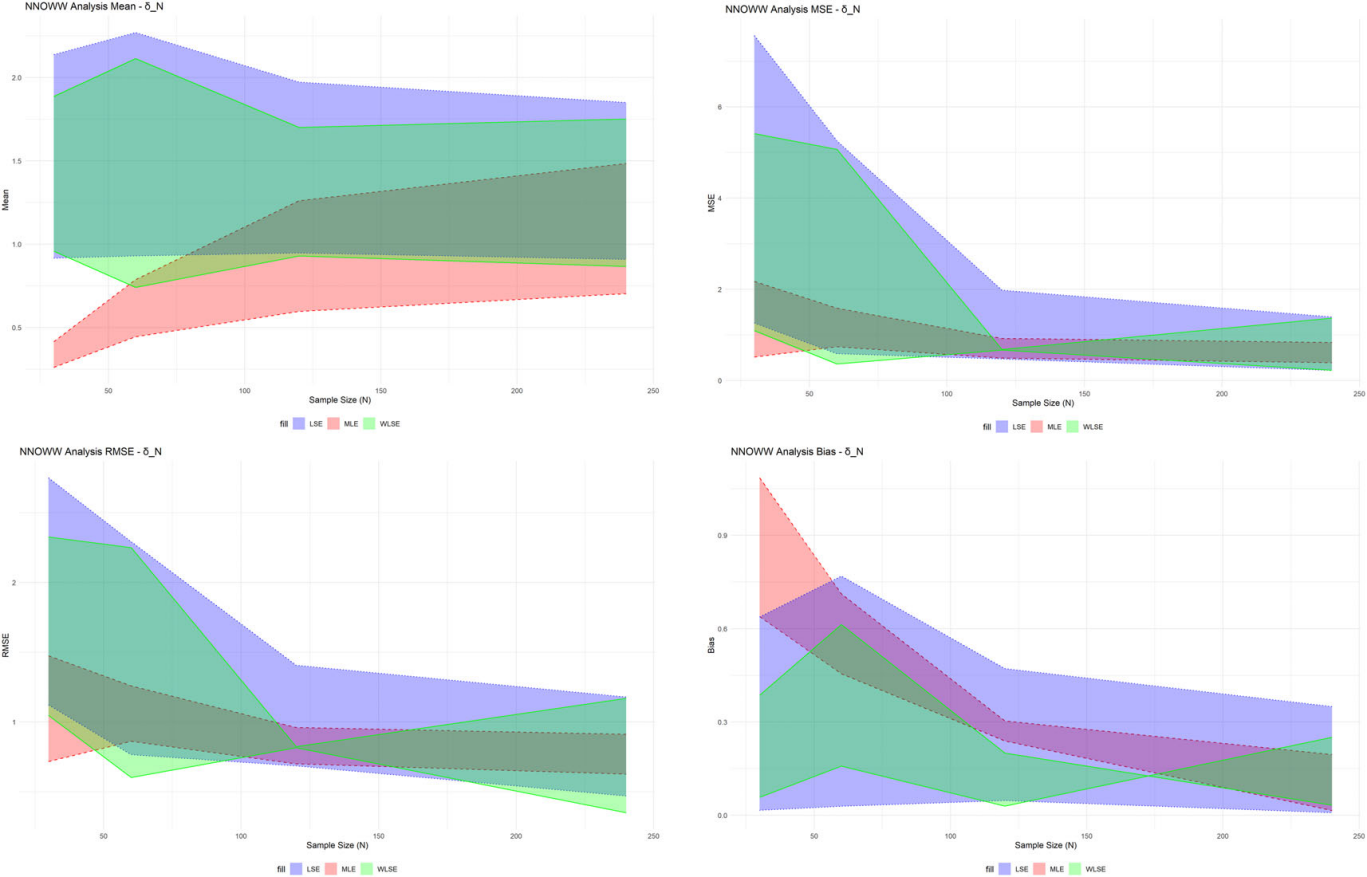

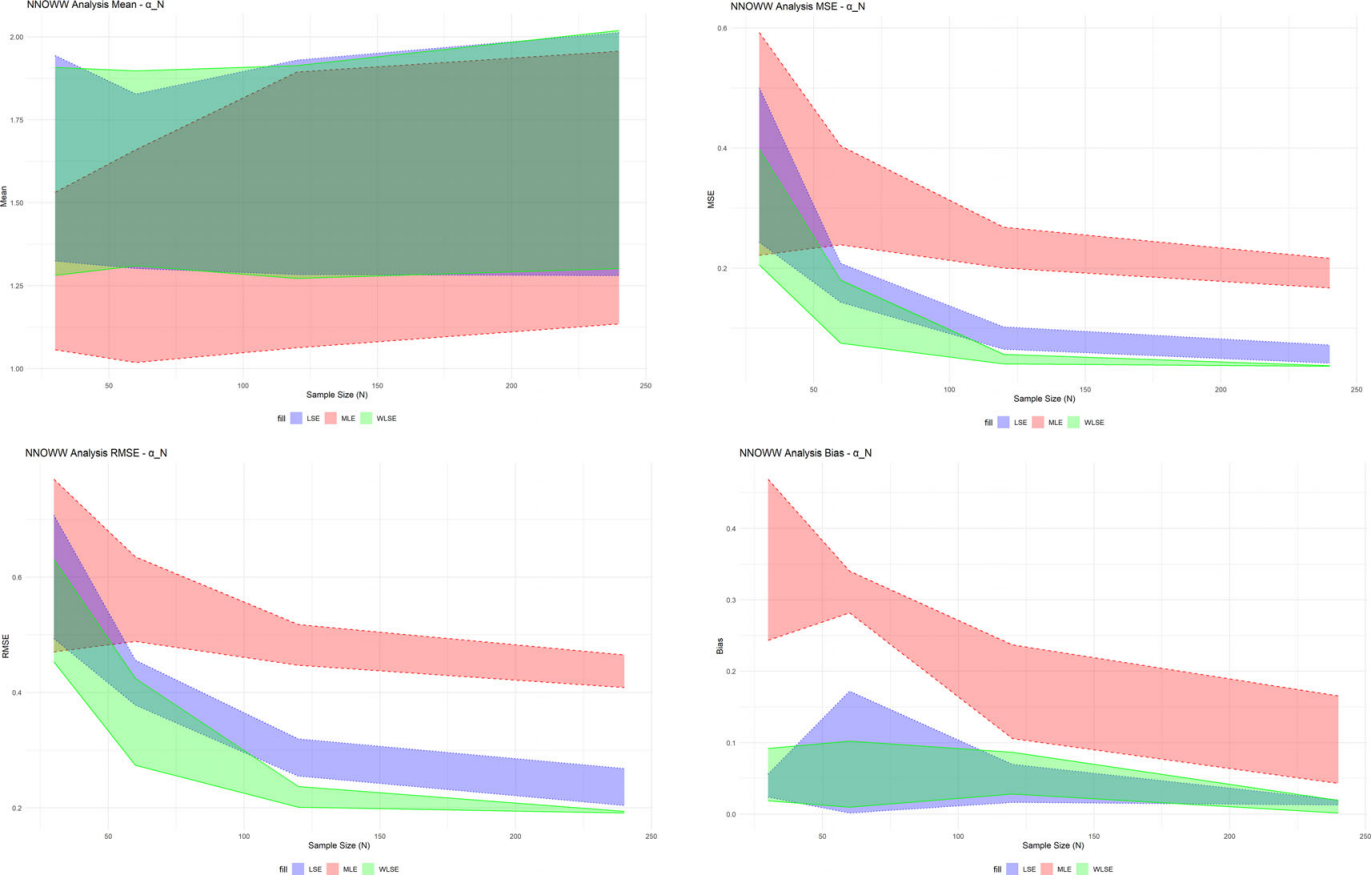

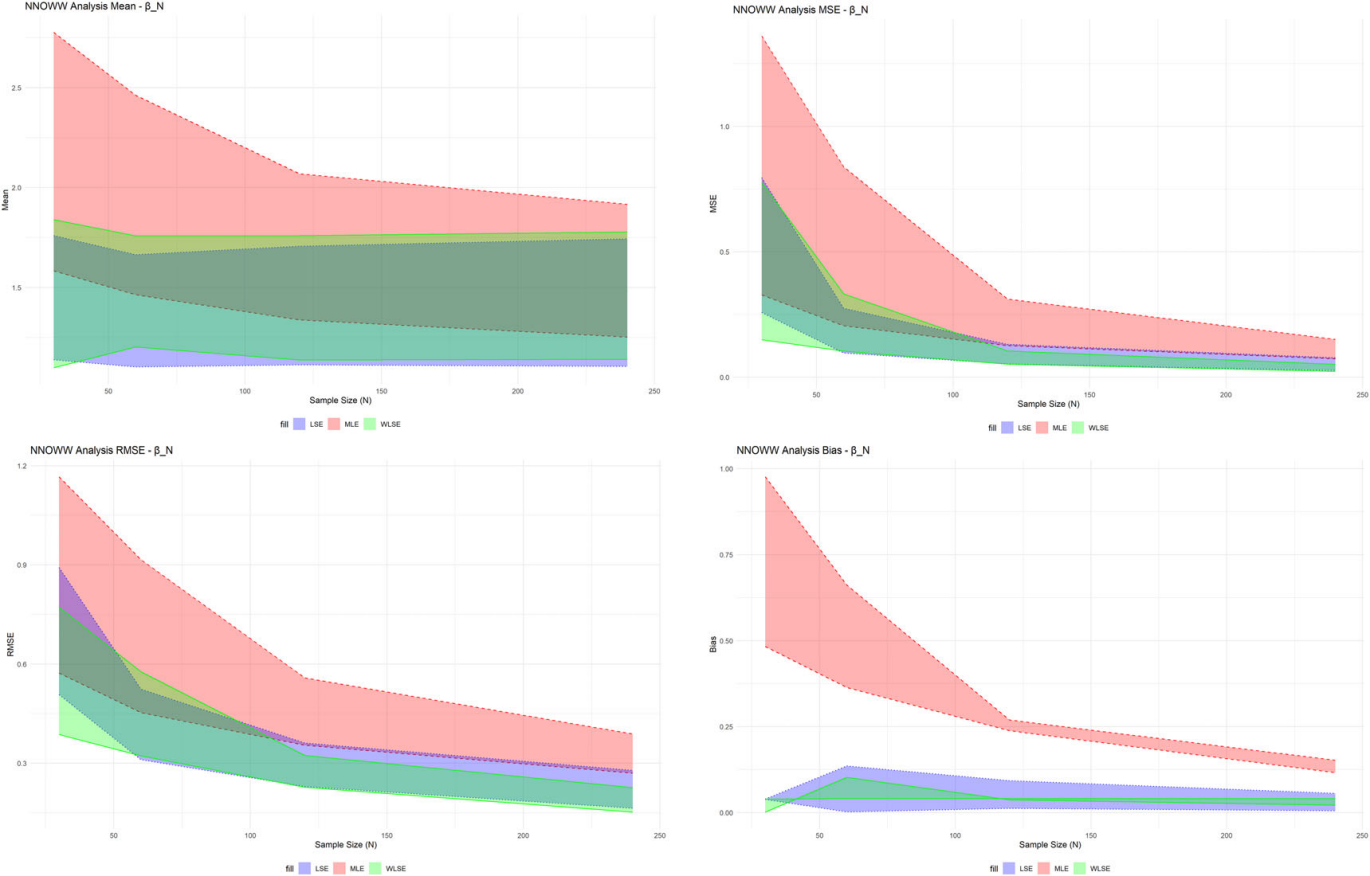

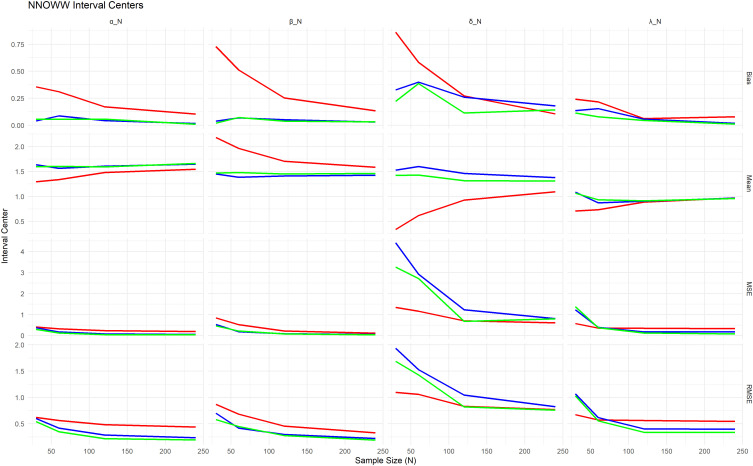

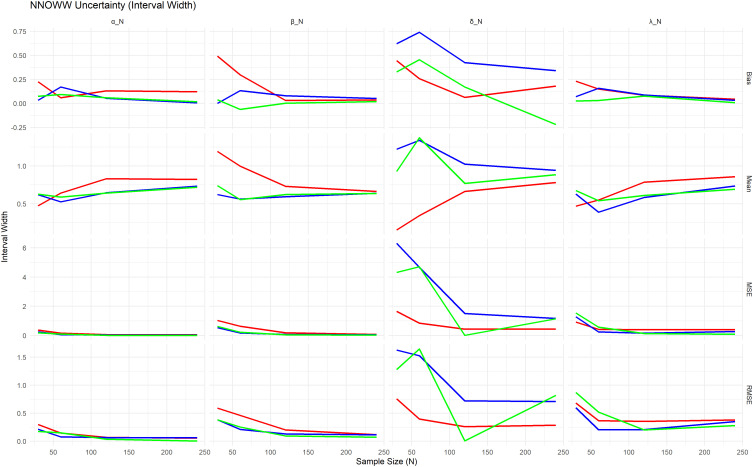
Draw simulation results.

Based on the simulation results in the table, the graphs in Figure 5 confirm the numerical results in the simulation table, providing a more precise visual representation.

The results show that when the sample size is small, the differences between methods are significant, and the estimates are unreliable. As the sample size increases, the differences narrow, and the estimates stabilize, especially for MLE.

For $\lambda_{N}$ , in small samples ( n=30 ), the lines appear scattered and wide, indicating high volatility and instability in the estimate, especially for LSE and WLSE. As the sample size increases, the lines begin to compress towards specific values, indicating improved accuracy and reduced dispersion. For the $\delta_{N}$ , the graphs show that WLSE significantly improved this estimate at sizes of 120 and 240 , and began to outperform LSE in reducing volatility. For $\alpha_{N}$ , this particular plot illustrates the parameters most significantly affected by the increase in the sample size at n=30 . The three lines are very far apart. At n=120 and n=240 , we find that the MLE approaches a clear horizontal line, indicating a quasi-stationary and highly accurate estimate. The WLSE gradually approaches but remains slightly fluctuating at the upper limit.

### Application on heart pulse count data

The applied part of the study focused on testing the effectiveness of the NNOWD by applying it to real-world data. The pulse rate is generally regarded as a discrete variable. The proposed test is applied using the pulse rate counts of 11 patients ^43^ was used as a real-world example to verify the suitability of the proposed distribution in describing data characterized by uncertainty. Before verifying the data, it is checked that the data meets the properties of neutral logic. Table 5 shows the actual values of the data (Intervals), the three neutral components (T, F, I), and the condition “ T+F+I=1 ” to verify the validity of the neutral formula.

**Table 5.** Data used, Truth, False, indeterminacy values and verification condition.

No.	Intervals	Truth (T)	Falsity (F)	Indeterminacy (I)	Sum (T+F+I)	Satisfy neutrosophic condition or not
1	[44, 68]	56	-24	-31	1	yes
2	[62, 72]	67	-10	-56	1	yes
3	[56, 90]	73	-34	-38	1	yes
4	[70, 112]	91	-42	-48	1	yes
5	[54, 72]	63	-18	-44	1	yes
6	[70, 100]	85	-30	-54	1	yes
7	[63, 75]	69	-12	-56	1	yes
8	[72, 100]	86	-28	-57	1	yes
9	[76, 98]	87	-22	-64	1	yes
10	[86, 96]	91	-10	-80	1	yes
11	[86, 100]	93	-14	-78	1	yes
12	[67, 88]	77.5	-21	-55.5	1	yes
13	[89, 100]	94.5	-11	-82.5	1	yes
14	[56, 65]	60.5	-9	-50.5	1	yes
15	[87, 93]	90	-6	-83	1	yes
16	[43, 70]	56.5	-27	-28.5	1	yes
17	[86, 112]	99	-26	-72	1	yes
18	[65, 77]	71	-12	-58	1	yes
19	[89, 99]	94	-10	-83	1	yes
20	[90, 101]	95.5	-11	-83.5	1	yes
21	[77, 89]	83	-12	-70	1	yes
22	[98, 115]	106.5	-17	-88.5	1	yes
23	[54, 66]	60	-12	-47	1	yes
24	[75, 89]	82	-14	-67	1	yes
25	[85, 100]	92.5	-15	-76.5	1	yes
26	[67, 78]	72.5	-11	-60.5	1	yes
27	[76, 97]	86.5	-21	-64.5	1	yes
28	[95, 117]	106	-22	-83	1	yes
29	[86, 99]	92.5	-13	-78.5	1	yes
30	[66, 100]	83	-34	-48	1	yes
31	[77, 88]	82.5	-11	-70.5	1	yes
32	[88, 99]	93.5	-11	-81.5	1	yes
33	[83, 112]	97.5	-29	-67.5	1	yes
34	[56, 65]	60.5	-9	-50.5	1	yes
35	[45, 78]	61.5	-33	-27.5	1	yes
36	[87, 97]	92	-10	-81	1	yes
37	[67, 89]	78	-22	-55	1	yes
38	[84, 102]	93	-18	-74	1	yes
39	[90, 109]	99.5	-19	-79.5	1	yes
40	[91, 108]	99.5	-17	-81.5	1	yes
41	[56, 77]	66.5	-21	-44.5	1	yes
42	[77, 99]	88	-22	-65	1	yes
43	[73, 89]	81	-16	-64	1	yes
44	[89, 114]	101.5	-25	-75.5	1	yes
45	[64, 70]	67	-6	-60	1	yes
46	[56, 74]	65	-18	-46	1	yes
47	[91, 117]	104	-26	-77	1	yes
48	[67, 88]	77.5	-21	-55.5	1	yes
49	[54, 77]	65.5	-23	-41.5	1	yes
50	[92, 100]	96	-8	-87	1	yes
Mean		82.67	-18.26	-63.41		
Sd-values		14.395156	8.282783	16.378087		
Max-values		106.5	-6.0	-27.5		
Min-values		56.0	-42.0	-88.5		

The following figures illustrate the data used, the nature of its analysis, and the components of its analysis.

All values satisfy the sum=1 condition, indicating the accuracy of the components’ calculations. Some negative values appear in F and I, indicating that the values fall outside the range [0,1] if no restriction or normalization is applied. However, the study analyzes them at the level of actual values, not relative values. The table reflects the methodology’s ability to extract the components of knowledge, ignorance, and uncertainty from real data.

All figures are used to validate the three components of neutrosophic logic (T, F, I) using graphs and to illustrate the behavior of these values across the data.

**Figure 6.**
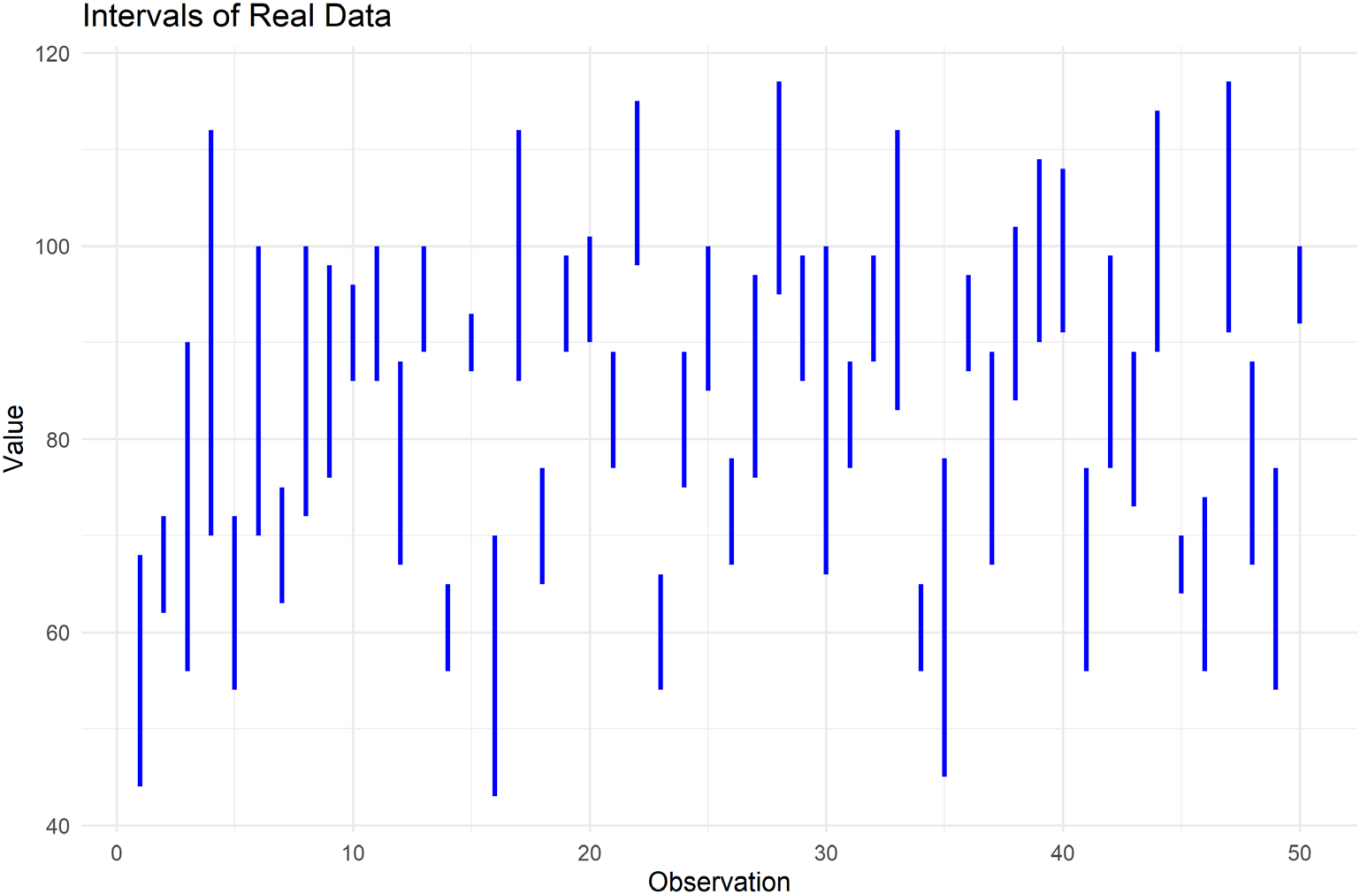
Plot the intervals for heart pulse count data.

Figure 6 plots the highest and lowest values for each observation, illustrating the range of data used in the T, F, I analysis.

**Figure 7.**
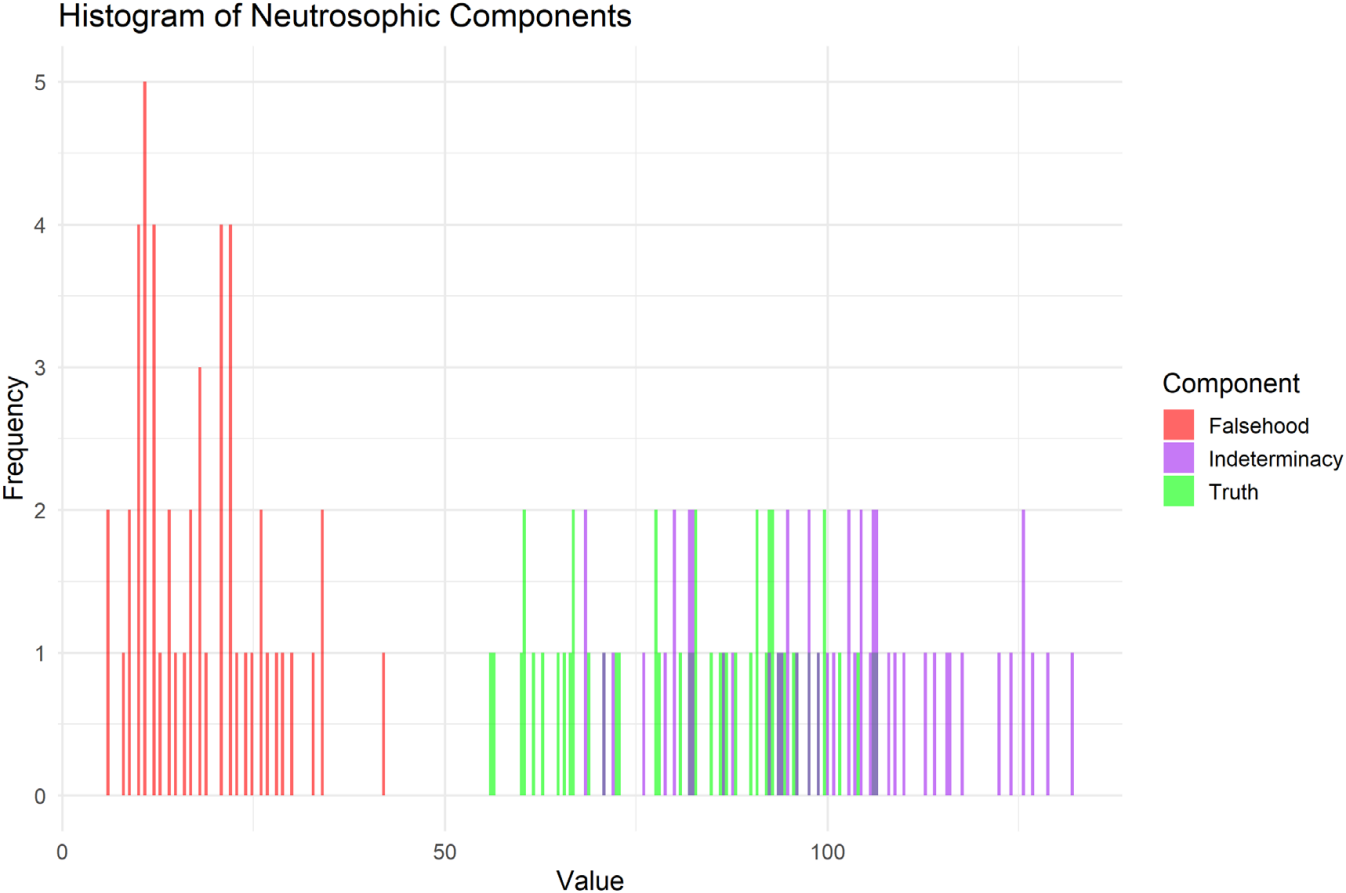
Histogram of neutrosophic components for heart pulse count data.

Figure 7 is a graphical representation of the distribution of T, F, and I across all observations, showing that the values are concentrated within a specific range for each component and illustrating the variance between them.

**Figure 8.**
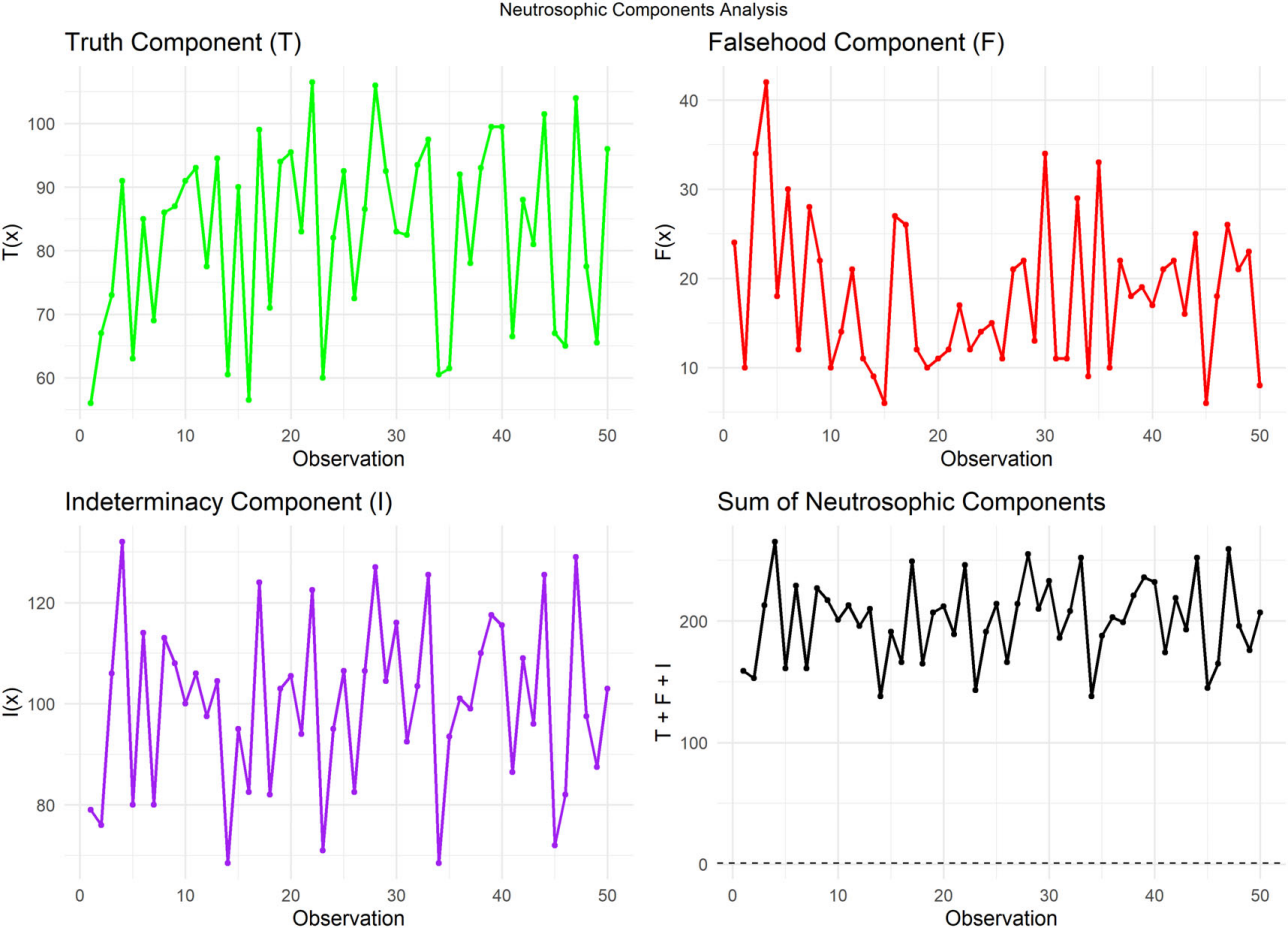
Neutrosophic parts and sum of neutrosophic components for heart pulse count data.

Figure 8 plots the sum of T, F, and I for each observation. This plot is a crucial step in validating the neutrosophic analysis, as it demonstrates that all values align with the value 1, a prerequisite for neutrosophic analysis. This validation ensures the accuracy and reliability of the methodology.

**Figure 9.**
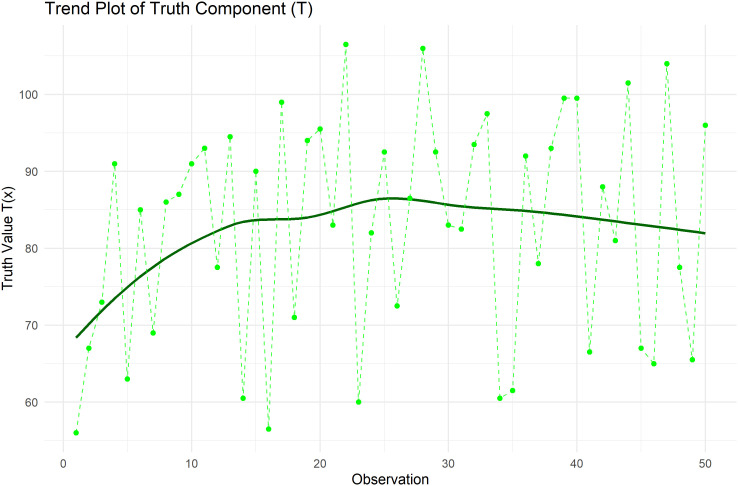
Trend plot of truth value for heart pulse count data.

Figure 9 plots Trend plot of Truth value for Heart Pulse Count Data.

After verifying the conditions of neutrosophic logic on the data used, the effectiveness of the proposed distribution is confirmed by comparing it with five other distributions because it’s have the same number of parameters, namely Neutrosophic Exponential Generalized Exponential Weibull (NEGW), Neutrosophic Kumaraswamy Weibull (NKuW), Neutrosophic Log-Gamma Weibull (NLGamW), Neutrosophic Beta Weibull (NBEW), and Neutrosophic Gompertz Weibull (NGoW). The comparison proceeds through three stages: first, using information criteria such as Akaike (AIC), ^44^ Corrected Akaike (CAIC), ^45^ Hann-Quinn (HQIC), ^46^ and Bayes criterion (BIC) ^47^; second, employing four statistical measures: Kolmogorov-Smirnov (KS), Anderson-Darling (A), Cramér-von Mises (W), and p-value; and finally, verifying the parameters estimated by the maximum likelihood method for each distribution. the analysis results which was shown in Table 6, Table 7 and Table 8.

**Table 6.** Information criteria for comparative distributions.

Dist.	-L	AIC	CAIC	BIC	HQIC
NNOWW	[204.2618, 205.6503]	[419.3006,416.5235]	[417.4124,420.1895]	[424.1716,426.9487]	[419.436, 422.2131]
NBEW	[207.2446, 208.1286]	[422.4892,424.2573]	[423.3781, 425.1462]	[430.1373, 431.9054]	[425.4016, 427.1697]
NKuW	[207.408, 207.9899]	[422.816, 423.9799]	[423.7049, 424.8687]	[430.4641, 431.6279]	[425.7284, 426.8923]
NEGW	[208.5483, 213.7341]	[425.0966,435.4682]	[425.9855, 436.3571]	[432.7447, 443.1163]	[428.009, 438.3806]
NLGamW	[206.3212, 218.4827]	[420.6423, 444.9655	[421.5312, 445.8543]	[428.2904, 452.6135]	[423.5548, 447.8779]
NGoW	[207.2046,251.2934]	[422.4091,510.6621]	[423.298,511.5509]	[430.0572,518.3101]	[425.3216,513.5745]

**Table 7.** Statistical measures for comparative distributions.

Dist.	W	A	K-S	p-value
NNOWW	[0.13974,0.14465]	[0.87114,0.89622]	[0.12684,0.14154]	[0.26907, 0.39696]
NBEW	[0.17807, 0.24427]	[1.20870, 1.3406]	[0.14198, 0.14959]	[0.21312,0.26572]
NKuW	[0.18018, 0.24436]	[1.22137, 1.3436]	[0.14345,0.14768]	[0.22553, 0.25487
NEGW	[0.19825], 0.26901]	[1.34247, 1.4651]	[0.13349, 0.16669	[0.12419,0.33500]
NLGamW	[17.41074,17.46944]	[99.70063, 99.73191]	[0.87094,0.98420]	0
NGoW	[0.13203],0.15718]]	[0.86941,1.05846]	[0.10851,0.46192]	[1.0822e-09,0.29807]

**Table 8.** Parameters for comparative distributions.

Dist.	$\lambda_{N}$	$\delta_{N}$	$\alpha_{N}$	$\beta_{N}$
NNOWW	[0.03036, 1.64019]	[3.81830,5.96502]	[0.00497,0.07416]	[0.76022, 1.20188]
NBEW	[28.50661, 28.66524]	[2.64882,5.46040]	[0.00865,0.02635]	[0.99591, 1.26557]
NKuW	[16.47589, 32.56959]	[1.81945, 2.18476]	[0.00795, 0.01329]	[1.21951,1.36260]
NEGW	[0.14701,0.17539]	[21.41174, 34.29560]	[0.04345, 0.14137]	[1.16102,1.43065]
NLGamW	[10.14389,14.33306]	[0.37516,0.55227]	[0.14272, 0.22895]	[1.06441,1.20872]
NGoW	[0.00321,0.91463]	[0.34233, 3.20060]	[0.00773, 0.02987]	[0.93344,1.13387]

Table 6 presents the statistical quality metrics for six neutrosophic distributions, including the proposed NNOWW distribution, and evaluates the effectiveness of each distribution. The results show that the NNOWW distribution has the lowest values on most of these criteria compared to the others, highlighting its superior data representation. Distributions such as NBEW, NKuW, and NEGW recorded higher values, indicating a poorer fit to the data. The NLGamW and NGoW distributions exhibited the highest values, demonstrating poor performance.

Table 7 includes the statistical test indicators for comparison. The NNOWW distribution records the lowest W and A values compared to the other distributions, indicating the best fit to the data. The highest p-value (between 0.269 and 0.396) suggests that the distribution is not rejected at a conventional significance level (0.05), which is not achieved by most other distributions. The NLGamW distribution recorded disastrous values (p = 0), indicating its complete failure to represent the data.

Table 8: Parameters estimated via MLE. It is clear that the NNOWW distribution has stable and moderate parameters, without extremes or excessive values, and exhibits stability and balance, which enhances its reliability.

**Figure 10.**
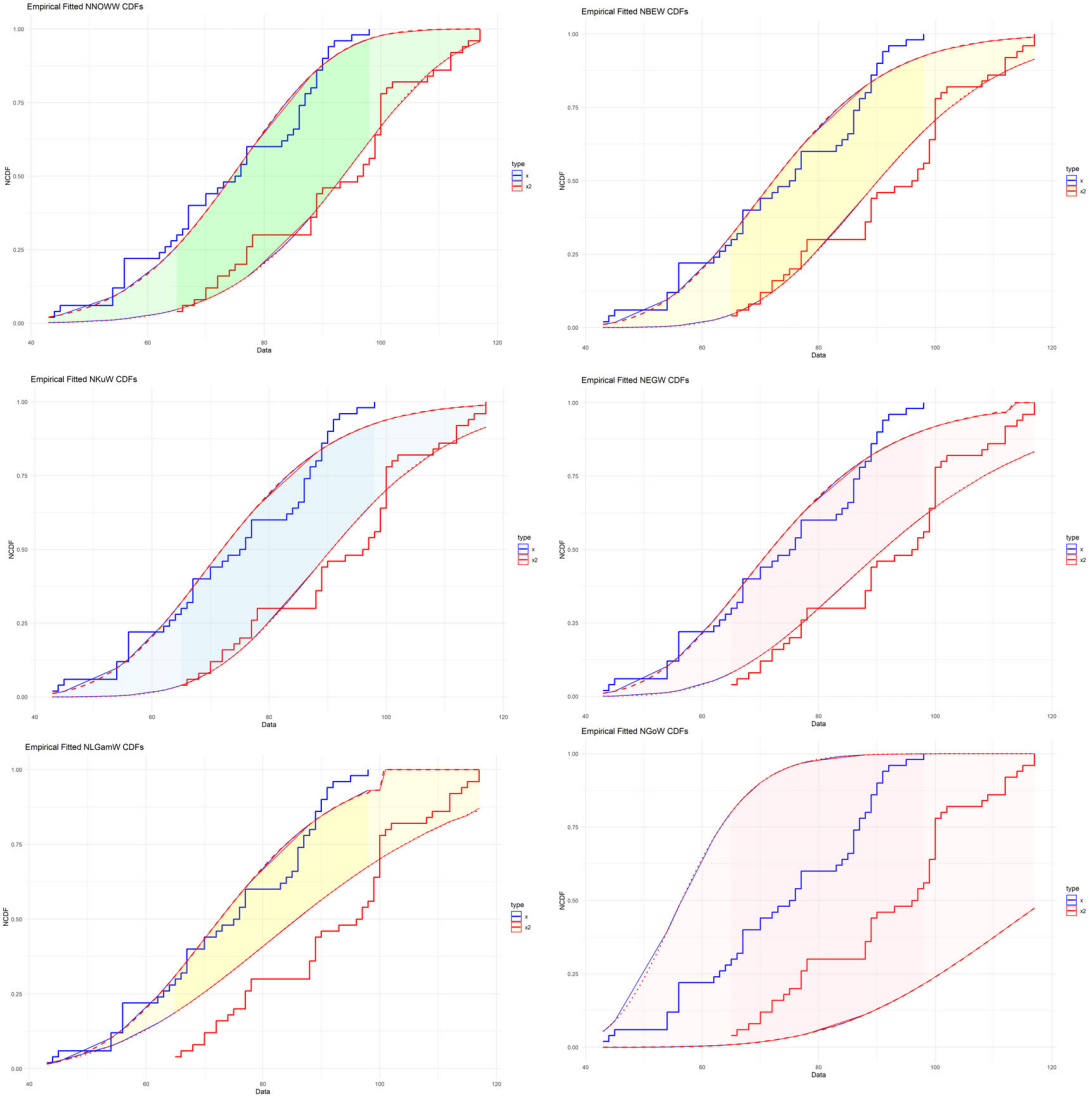
Empirical NCDF for comparative distributions.

To confirm what was presented in Tables 6, 7 and 8, Figure 10 is given as follows:

The Figure 10 compares the true NCDF with estimates from various models. The NNOWW distribution curve closely matches the curve from the experimental data. The other models show deviations in several areas, especially at the extremes where the variances increase, impacting the distribution’s reliability. This visual clearly supports what the tables indicate: the NNOWW distribution most accurately represents the data.

## Conclusions

The NNOWW distribution provides a flexible mathematical structure by deriving density, cumulative, survival, and hazard functions in neutrosophic form. Moments, variance, skewness, and kurtosis are formulated using extended integrals, providing precise tools for analyzing neutrosophic data. The distribution also demonstrated the ability to contain indeterminacy and ambiguity via the NQF and standard deviation. The variability and overlap of parameters within non-sharp domains support a realistic representation of uncertain data. The distribution is suitable for analyzing data with atypical characteristics. Simulations were conducted using samples of different sizes to evaluate the performance of three estimation methods. The results indicate that NNOWW is capable of generating data that is close to reality and exhibits good statistical stability and consistency. The graphs confirmed the visual improvement in the reliability of the estimates as the sample grew. These results demonstrate the effectiveness of NNOWW as an accurate representation tool in statistical settings with ambiguous data. The NNOWW distribution was applied to heart rate data from 50 patients in a neutrosophic setting using the (T, F, I) components. All data met the neutrosophic condition ( T+F+I=1 ), proving its validity for analysis. The model results showed a high accuracy in representing the proper distribution of the data compared to five other neutrosophic distributions. The experimental shape of the cumulative function showed that NNOWW closely approximated the curve of the actual data, confirming its good fit. The model provided an accurate representation of data with ambiguity or uncertainty, such as in biometric measurements.

## Data Availability

The data used in this study were obtained from a third-party source and were not generated by the authors. The underlying data consist of interval-valued heart pulse count measurements originally published by Gioia and Lauro (2005) in “Basic statistical methods for interval data”, Statistica Applicata, 17(1), 75–104, or at the following link: https://osf.io/8xa4t/overview. The authors do not have the right to redistribute these data in a separate repository. However, readers and reviewers can access the same dataset directly from the original published source using the same procedures followed by the authors. Additional supporting materials related to the pulse oximeter device, including design files and documentation, are publicly available through the Open Science Framework at DOI: https://doi.org/10.17605/OSF.IO/8XA4T.
