# Supplementary material for: Neutrosophic New Odd Weibull-Weibull Distribution with Heart Pulse Count Data Application
Source: F1000Res. 2026 Mar 28;15:222. Originally published 2026 Feb 10. [Version 2] doi: 10.12688/f1000research.172591.2 (PMC13096788; doi:10.12688/f1000research.172591.2)
Supplement: Peer review history

# Reviewer report: Reviewer response for version 2

**DOI:** 10.5256/f1000research.197571.r471668

**Published:** 2026-04-20

**Authors:** Hadi Obaid Alshammari

**Affiliations:** 1 Jouf University, Sakaka, Saudi Arabia

This manuscript introduces the Neutrosophic New Odd Weibull-Weibull (NNOWW) distribution as an extension of the New Odd Weibull-Weibull family within a neutrosophic framework for uncertain data. The paper develops the main distributional properties, including the cumulative distribution function, density, survival and hazard functions, quantile function, moments, incomplete moments, characteristic and moment generating functions, and Rényi entropy. The authors also study parameter estimation using MLE, LSE, and WLSE, provide a Monte Carlo simulation study for several sample sizes, and apply the model to heart pulse count data from 50 patients with comparison against competing neutrosophic models.  

 After carefully reading the revised version, I find that the manuscript has improved notably since the first version. Several issues previously raised by reviewers appear to have been addressed, particularly the consistency of notation, clarification of the simulation purpose, and the interpretation of the real-data comparison results. The revised manuscript is more coherent and easier to follow.

 The topic is relevant within the growing literature on generated distributions and neutrosophic statistics. The proposed model appears flexible, and the real-data example suggests that it performs competitively when compared with the alternative models considered by the authors. The paper is also well structured overall, moving naturally from model construction to properties, estimation, simulation, and application.

 In my opinion, the manuscript is suitable for indexing after minor revisions. My comments below are intended mainly to improve presentation and reproducibility.

 Comments:

 1- The mathematical development is substantial, but the formulas in the section dealing with expansions (especially Equations (11)–(14)) remain quite dense. I suggest a small improvement in formatting so that the coefficients and summation terms are easier to read. Breaking some long expressions into multiple lines would help future readers.

 2- The estimation procedures are described mathematically, but the paper would benefit from one short sentence stating which software or numerical package was used for optimization and simulation. This would improve reproducibility.

 3- The manuscript reads much better than before, but a final light proofreading would still be beneficial. A few sentences could be smoothed stylistically for stronger academic flow.

Is the work clearly and accurately presented and does it cite the current literature?

Yes

If applicable, is the statistical analysis and its interpretation appropriate?

Yes

Are all the source data underlying the results available to ensure full reproducibility?

Partly

Is the study design appropriate and is the work technically sound?

Yes

Are the conclusions drawn adequately supported by the results?

Yes

Are sufficient details of methods and analysis provided to allow replication by others?

Partly

Reviewer Expertise:

Functional Analysis, Operator Theory, Mathematical Analysis, , Hilbert Space Theory, Banach Space Theory, Spectral Theory, Linear Operators, Inequalities, Mathematical Modelling, Mathematical Statistics.

I confirm that I have read this submission and believe that I have an appropriate level of expertise to confirm that it is of an acceptable scientific standard.

# Reviewer report: Reviewer response for version 2

**DOI:** 10.5256/f1000research.197571.r471357

**Published:** 2026-04-07

**Authors:** Eslam Hussam

**Affiliations:** 1 Prince Sattam bin Abdulaziz University, Al Kharj, Riyadh Province, Saudi Arabia

I Accept the revised version

Is the work clearly and accurately presented and does it cite the current literature?

Yes

If applicable, is the statistical analysis and its interpretation appropriate?

Yes

Are all the source data underlying the results available to ensure full reproducibility?

Yes

Is the study design appropriate and is the work technically sound?

Yes

Are the conclusions drawn adequately supported by the results?

Yes

Are sufficient details of methods and analysis provided to allow replication by others?

Yes

Reviewer Expertise:

Statistics

I confirm that I have read this submission and believe that I have an appropriate level of expertise to confirm that it is of an acceptable scientific standard.

# Reviewer report: Reviewer response for version 1

**DOI:** 10.5256/f1000research.190330.r458871

**Published:** 2026-03-05

**Authors:** Hamza Abubakar

**Affiliations:** 1 Isa Kaita College of Education, Dutsin-Ma, Katsina, Nigeria

This paper introduces the Neutrosophic New Odd Weibull-Weibull (NNOWW) distribution, which combines the New Odd Weibull-Weibull distribution with neutrosophic logic to handle uncertain and imprecise data. The authors derive key statistical functions including the cumulative distribution function, probability density function, survival function, and hazard function in neutrosophic form. They develop mathematical properties such as moments, quantile function, variance, skewness, kurtosis, and Rényi entropy. Parameter estimation is performed using three methods—Maximum Likelihood Estimation, Least Squares Estimation, and Weighted Least Squares Estimation—with Monte Carlo simulations across sample sizes of 30, 60, 120, and 240. The model is applied to heart pulse count data from 50 patients and compared against five competing neutrosophic distributions using multiple information criteria and statistical tests. The authors conclude that NNOWW demonstrates superior performance in representing uncertain medical data.

 The paper contains several fundamental technical errors that must be addressed before it can be considered scientifically sound. First, the log-likelihood function in Equation (31) is misspecified, incorrectly including `nlog(λN)` when λN should appear in an exponent term. This error affects all subsequent MLE-based results in Tables 4 and 8 and requires complete recalculation. Second, multiple tables present intervals with invalid ordering where lower bounds exceed upper bounds—notably in Table 2 (variance, skewness, and kurtosis values) and Table 6 (AIC criteria). The AIC values in Table 6 are specifically reversed, with the lower bound corresponding to the upper -L value. Third, Table 5 presents neutrosophic components with negative values (e.g., F=-42, I=-88.5) without justification, violating standard neutrosophic theory where components should be non-negative. The paper must either justify negative values, transform the data, or clarify what these values represent.

 Fourth, Equation (14) contains circular reasoning by including the parameter δN within summation indices, making the expansion dependent on the very parameter being estimated. Fifth, Table 7 presents p-values as intervals without any explanation of their interpretation, particularly problematic for NGoW where the interval spans nine orders of magnitude. Sixth, there is a fundamental disconnect between simulation parameters (αN=[1.3,2], δN=[0.9,1.5]) and real data estimates (αN=[0.00497,0.07416], δN=[3.81830,5.96502]), rendering the simulation results potentially irrelevant to the application. Seventh, Table 3 reports mathematically impossible negative "average interval" widths and miscalculated values that must be corrected.

 Beyond the critical issues, several improvements would strengthen the paper. The notation for parameters versus summation indices needs clarification throughout Equations (11-14). The discussion of small-sample performance in the simulation underestimates the severity of bias, particularly for δN at n=30. Sensitivity analysis for the wide parameter intervals in the real data application would help assess model stability. The series expansion in Equation (14) requires convergence verification. All figures need replacement with high-resolution vector graphics and expanded captions. Given the fundamental nature of the errors identified—particularly the misspecified likelihood function and invalid interval presentations—the manuscript cannot be accepted in its current form. However, the topic has merit, and with careful revision addressing all issues raised, the paper could make a valuable contribution to the literature on neutrosophic distributions. The recommendation is therefore to reject with the option to resubmit after major revisions, requiring a point-by-point response and clear indication of all changes made.

Is the work clearly and accurately presented and does it cite the current literature?

No

If applicable, is the statistical analysis and its interpretation appropriate?

No

Are all the source data underlying the results available to ensure full reproducibility?

Partly

Is the study design appropriate and is the work technically sound?

Partly

Are the conclusions drawn adequately supported by the results?

No

Are sufficient details of methods and analysis provided to allow replication by others?

Partly

Reviewer Expertise:

Probability and Distribution Theory

I confirm that I have read this submission and believe that I have an appropriate level of expertise to confirm that it is of an acceptable scientific standard, however I have significant reservations, as outlined above.

# Response

**Published:** 2026-03-09

**Authors:** Nooraldeen Alsaab

**Affiliations:** Mathematics, Anbar General Directorate of Education, Ministry of Education, Fallujah, Iraq

Dear Reviewer,

 We sincerely thank you for your detailed and insightful review. Your comments were extremely valuable and helped us improve both the technical correctness and clarity of the manuscript. All issues raised have been carefully addressed in the revised version. The main revisions are summarized below.

**Comment 1 – Log-likelihood specification**

 The log-likelihood expression in Equation (31) has been carefully re-derived and corrected. The term involving λN\lambda_NλN​ has been revised so that the exponent δN\delta_NδN​ is correctly included in the likelihood structure.

**Comment 2 – Invalid interval ordering in tables**

 All neutrosophic intervals reported in the manuscript have been rechecked. The tables were corrected to ensure that each interval follows the correct ordering

 lower < upper.

 In particular, the information criteria values in Table 6 and the statistical measures in Table 2 were carefully verified and corrected where necessary.

**Comment 3 – Negative interval widths**

 Table 3 has been recalculated. The average interval width is now computed as

 width = upper − lower

 ensuring that all values are positive and consistent with the definition of interval width.

**Comment 4 – Series expansion formulation**

 The series expansion used in the derivation of the density representation was reformulated to ensure that summation indices are independent of the model parameters. The notation of the coefficients ψ\psiψ and ϕ\phiϕ was also clarified.

**Comment 5 – Interpretation of interval p-values**

 The explanation of interval-valued p-values has been expanded to clarify their interpretation in the neutrosophic statistical framework.

**Comment 6 – Simulation versus real-data parameters**

 A clarification paragraph has been added in the Monte Carlo simulation section explaining that the simulation parameters were selected to represent general distributional behavior rather than to reproduce the exact scale of the real dataset.

**Comment 7 – Verification of tables and numerical results**

 All numerical tables were re-checked and corrected where necessary to ensure internal consistency and computational accuracy.

 We are grateful for the reviewer’s careful evaluation, which substantially strengthened the technical rigor of the manuscript.

# Reviewer report: Reviewer response for version 1

**DOI:** 10.5256/f1000research.190330.r458866

**Published:** 2026-02-24

**Authors:** Eslam Hussam

**Affiliations:** 1 Prince Sattam bin Abdulaziz University, Al Kharj, Riyadh Province, Saudi Arabia

Reviewer Report

 The manuscript proposes a new neutrosophic extension of the New Odd Weibull-Weibull distribution and provides comprehensive theoretical derivations, simulation analysis, and an applied medical data example. The mathematical development is extensive and well-structured. The Monte Carlo study adequately evaluates estimator performance, and the real data application supports the theoretical findings.

 The contribution is relevant within the field of neutrosophic statistics and generated distribution families. The results indicate clear superiority of the proposed model according to standard information criteria.

 I recommend acceptance after minor revisions addressing the following points:

 1.    Improve clarity and formatting of some series expansion formulas and notation consistency. Some equations in Series Expansions need to be reformatted to be clearer, especially in the definition of the coefficients ψ and φ.

 2.    Add a brief paragraph in the introduction clarifying the specific added value of the neutrosophic framework compared to classical interval estimation. What is the difference between classical interval estimation and the philosophy of neutrosophy? And why is the neutrosophy framework specifically suitable for heart rate data?

 3.    Strengthen the interpretation of Monte Carlo results by explicitly highlighting convergence behavior as sample size increases. 

 4.    The Monte Carlo results are well presented in the tables, but the explanation could be more focused. A short paragraph clearly stating that the MLE is the most statistically stable across all sample sizes would be beneficial.

 5.    Expand slightly the discussion of information criteria differences in the real-data application. Comparing using information criteria is excellent, but it would be preferable to add a sentence explaining why the difference in AIC/BIC is considered practically significant.

 6.    There are some minor stylistic errors in the English phrasing. A light, professional proofreading is recommended to improve the academic flow of the text. Examples include: 

 i.    Classical continuous distributions data back to the 18th century; 

 ii.    This development has become evident, particularly with the emergence of new mathematical concepts that address uncertainty and ambiguity in data; 

 iii.    such data derived from medical measurements or observational studies, such as those derived from medical experiments or environmental measurements.

 After addressing these points, the manuscript will be suitable for indexing.

Is the work clearly and accurately presented and does it cite the current literature?

Yes

If applicable, is the statistical analysis and its interpretation appropriate?

Yes

Are all the source data underlying the results available to ensure full reproducibility?

Yes

Is the study design appropriate and is the work technically sound?

Yes

Are the conclusions drawn adequately supported by the results?

Yes

Are sufficient details of methods and analysis provided to allow replication by others?

Yes

Reviewer Expertise:

Statistics

I confirm that I have read this submission and believe that I have an appropriate level of expertise to confirm that it is of an acceptable scientific standard, however I have significant reservations, as outlined above.

# Response

**Published:** 2026-03-09

**Authors:** Nooraldeen Alsaab

**Affiliations:** Mathematics, Anbar General Directorate of Education, Ministry of Education, Fallujah, Iraq

Dear Reviewer,

 We sincerely thank you for your careful reading of the manuscript and for your constructive comments, which helped us improve the clarity and presentation of the paper. All suggested revisions have been incorporated in the revised manuscript using the track-changes feature. The main modifications are summarized below.

**Comment 1 – Clarity of series expansions and notation**

 The equations related to the series expansions have been reformatted and the notation of the coefficients ψ\psiψ and ϕ\phiϕ has been revised to improve readability and consistency. The mathematical expressions were rewritten and the summation indices were clarified to avoid ambiguity.

**Comment 2 – Clarifying the added value of neutrosophic framework**

 A new explanatory paragraph has been added in the Introduction to clarify the conceptual difference between classical interval estimation and the neutrosophic framework. The text now explicitly explains that neutrosophic statistics models uncertainty using the triplet (truth, falsity, indeterminacy), which allows a richer representation of imprecision in medical measurements such as heart pulse data.

**Comment 3 – Interpretation of Monte Carlo convergence**

 The Monte Carlo section has been expanded to explicitly explain the convergence behavior of the estimators as the sample size increases. Additional explanatory text now highlights the reduction in bias and MSE as the sample size grows.

**Comment 4 – Emphasis on the stability of MLE**

 A new paragraph has been added to the simulation results section explaining that the Maximum Likelihood Estimator demonstrates the most stable statistical behavior across all sample sizes compared with the alternative estimation methods.

**Comment 5 – Interpretation of AIC/BIC differences**

 The discussion of the real data application has been expanded to clarify why the differences in AIC, BIC, CAIC, and HQIC indicate meaningful practical improvement of the proposed model relative to competing distributions.

**Comment 6 – Stylistic and language corrections**

 Several sentences in the Introduction and throughout the manuscript have been edited to improve clarity and academic flow. Minor grammatical and stylistic issues have been corrected.

 We appreciate the reviewer’s valuable suggestions, which significantly improved the clarity and presentation of the manuscript.
